# Restrained Wnt Signaling Pathway by Enhanced EsGSK3β Activity Facilitates the Infection of *Spiroplasma* and Leads to Neuropathic Diseases in Crustaceans

**DOI:** 10.1016/j.mcpro.2025.101059

**Published:** 2025-08-25

**Authors:** Libo Hou, Yu Yao, Yubo Ma, Wei Gu, Wen Wang, Wei Sun, Qingguo Meng

**Affiliations:** 1 Jiangsu Key Laboratory for Aquatic Crustacean Diseases, College of Marine Science and Engineering, Nanjing Normal University, Nanjing, China; 2 Engineering Lab of Henan Province for Aquatic Animal Disease Control, College of Fisheries, Henan Normal University, Xinxiang, China; 3 Department of Disinfection and Vector Control, Jiangsu Provincial Center for Disease Control and Prevention, Nanjing, Jiangsu, China

**Keywords:** Wnt signaling pathway, intracellular bacterium, crustacean, neurological disease, innate immunity

## Abstract

*Spiroplasma eriocheiris* has been identified as a lethal pathogen of *Eriocheir sinensis* tremor disease (TD), one neurological disease with typically paroxysmal tremors of the pereiopod. But, the pathogenic mechanism of TD was still unknown. First, in the current study, the phosphoproteomic changes of *E. sinensis* thoracic ganglion after *S. eriocheiris* infection were obtained using TMT labeling and affinity enrichment followed by high-resolution LC-MS/MS analysis. A total of 349 phosphorylation proteins are upregulated, and 331 phosphorylation proteins are downregulated when compared to the control sample with quantitative ratios above 1.5 or below 1/1.5 that are deemed significant. Bioinformatics analysis (Gene ontology, Kyoto Encyclopedia of Genes and Genomes pathway, and domain analysis, etc.) showed that Wnt signaling pathways were restrained, corresponding to many nervous system developments, and signal transmission pathway was also disrupted. Neurotransmitter metabolite analysis showed metabolic dysregulation of four key neurotransmitters (5-Hydroxy-L-tryptophan, serotonin, acetylcholine, and γ-amino-butyric acid) in thoracic ganglion of *E. sinensis* following *S. eriocheiris* infection. Second, similar to its effects on the thoracic ganglion cells, *S. eriocheiris* infection of hemocytes (the primary target cells and most crucial immune cells in crabs) also suppressed the host Wnt pathway through enhanced EsGSK3β activity, both *in vivo* and *in vitro*. Co-immunoprecipitation analysis showed that EsGSK3β could directly interact with Es*β*-catenin. Inhibited or enhanced EsGSK3β activity of hemocytes could reduce or facilitate *S. eriocheiris* infection by regulating the stability and nuclear translocation of Es*β*-catenin. Finally, further analysis showed that Wnt-*β*-catenin pathway could functionally crosstalk with Toll pathway to positively regulate hemocytes antibacterial peptides transcription. Altogether, our results suggest that *S. eriocheiris* could restrain the crab Wnt pathway, reduce antibacterial peptides transcription to help its infection and disorder neurotransmitters to cause neuropathic TD at last.

## Highlights

• *S*. *eriocheiris* caused crustacean neuropathic diseases by disorder Wnt pathway.
• *S. eriocheiris* restrain host hemocytes Wnt-*β*-Catenin pathwayhelp itself infection.
• The crab Wnt-*β*-Catenin pathway crosstalk with Toll pathway regulates AMPs expression.

## Graphical Abstract

**Figure:**
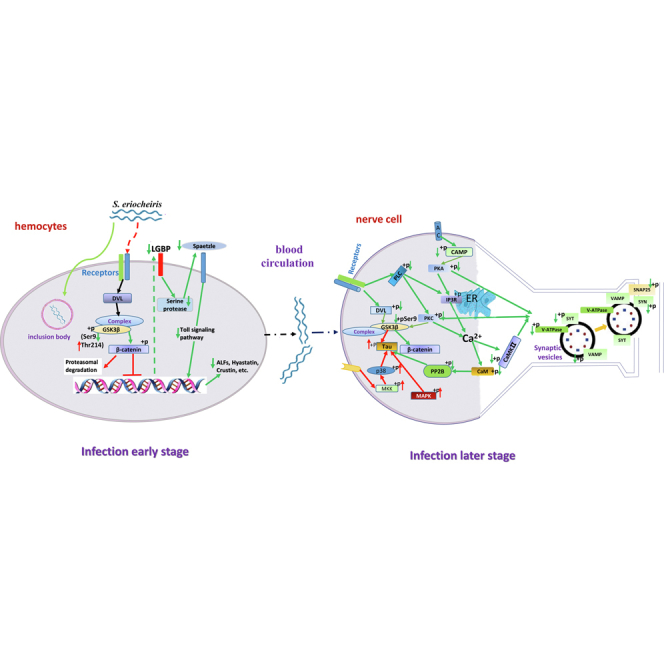

## In Brief

The present study combines the phosphoproteomic quantitative analysis, neurotransmitter metabolite analysis, transcriptomics analysis, and molecular biology experiments and revealed that the Wnt pathway plays a critical role in *S. eriocheiris* infection and causes neuropathic diseases in crustaceans. This is the first report about the molecular mechanisms of neurogenic disease in crustaceans caused by intracellular bacteria. Our results in this article provide novel insight to help us understand the relationship between the Wnt pathway in crustaceans and neurological disease caused by intracellular bacteria.

The Wnt pathway plays an important role in a variety of biological processes, including cell specification, cell polarity, and embryonic patterning. More evidence supports that Wnt signaling controls neuronal differentiation, dendrite development, and synaptic function and regulates the function and formation of neurons in the nervous system (1, 2). To date, many studies have established that dysregulation of the Wnt pathway contributes significantly to the development of major neurodegenerative disorders such as Huntington’s disease, Alzheimer’s disease (AD), and Parkinson’s disease (3, 4). However, there has been little research on neuropathic diseases in crustaceans. Tremor disease (TD), with typically paroxysmal tremors of the pereiopod, is one of the most serious neurogenic diseases of freshwater crustaceans (5). *Spiroplasma*, one of the smallest prokaryotes and a facultative intracellular pathogens, has been identified as a novel causative pathogen of TD in crustaceans and given the name *Spiroplasma eriocheiris* (6, 7). In recent years, infections caused by this bacterium have resulted in major economic losses in China's freshwater aquaculture industry (8, 9).

*Spiroplasma*, a member of Mollicute class, exhibits unique neurotropic characteristic. For example, *Spiroplasma mirum*, originally isolated from the rabbit tick (*Hemaphysalis leporispalustris*), is capable of infecting vertebrates and inducing the presence of a suckling mouse cataract agent (10). Similarly, *S. eriocheiris* has been detected in the brain of embryonated chickens (11) and can infect neonatal mice and cause cataracts (12). In freshwater crustaceans, hemocytes serve as the primary cellular targets for *S. eriocheiris* infection. Following invasion, *S. eriocheiris* can form inclusion bodies within hemocytes. Subsequently, through hematogenous dissemination, this pathogen can infect various host tissues, including muscles, nerves, and connective tissues (13). As the target organ, the thoracic ganglion infection by *S. eriocheiris* is directly responsible for the manifestation of TD. However, the molecular mechanism of paroxysmal tremors caused by *S. eriocheiris* is not clear.

In addition to its well-characterized role in nervous system development, the Wnt pathway is increasingly recognized as a crucial regulator of the hosts innate immunity through direct regulation or crosstalk with other pathways. The Wnt-*β*-catenin pathway participates in regulating the inflammatory response by crosstalk with the NF-кB signaling pathway in a mouse model against *Salmonella typhimurium* infection (14). However, compared with vertebrates, the immunological functions of Wnt pathway in crustacean remain poorly characterized and lack systematic investigation. For example, in *Litopenaeus vannamei*, Wnt-*β*-catenin pathway can stimulate expression of several antimicrobial peptides (AMPs) and thereby contributes to host defense against invading pathogens, but the underlying mechanism remains elusive (15). From the above, the Wnt pathway has been shown to play an important role both in the development of neurodegenerative disorders and innate immunity regulation. However, as *S. eriocheiris* is a special kind of intracellular bacterium, the specific roles of the Wnt pathway in the development of *S. eriocheiris*-induced neurogenic disease and host cell response against this pathogen infection in crustaceans remain completely unexplored.

To elucidate the detailed mechanisms of the Wnt pathway in both neurological diseases and host cell responses during *S. eriocheiris* infection in crustaceans, we used *S. eriocheiris*-infected Chinese mitten crab to cause TD as an infection model. In the present study, we found that the Wnt pathway played a pivotal role in *S. eriocheiris* infection and the development of subsequent neuropathic diseases in crabs. This report is the first study on neurogenic disease caused by an intracellular bacterium in crustaceans and the first systematic study of the role of the Wnt pathway in response to intracellular bacterial infection.

## Experimental Procedures

### Ethical Statement

The animal use and care protocols were approved by the Institutional Animal Care and Use Committee of Nanjing Normal University [SYXK (Jiangsu) 2020-0047 and IACUC-20220258]. All methods were performed in accordance with the relevant guidelines and regulations. No special sampling permission was required.

### Experimental Bacterial Infection and Thoracic Ganglion Collection

The *S. eriocheiris* used in this study was isolated from the diseased *Eriocheir sinensis* following the methods described by Wang *et al*. (13) and was cultured in R2 medium (heart infusion broth cultivation, 25 g/L; sucrose, 8 g/L; and PBS-B, 8 ml/L) at 30 °C. Crabs (50 ± 5 g) were purchased from an aquaculture pond in Baoying, Jiangsu Province, China, and cultivated in an ultraviolet radiation sterilized, temperature-controlled, and circulating aquaculture system. Healthy crabs (verified by *S. eriocheiris-*negative results using PCR by 16S rDNA sequence analyses) were maintained for 1 week before tests. The crabs in the experimental group (50 individuals cultivated in five tanks) received an injection of 100 μl of *S. eriocheiris* (10^8^ cells/ml), individually. Fifty crabs (cultivated in five tanks) in the control group received an injection of 100 μl of R2 medium. Ten crabs were randomly selected (two crabs were selected randomly from each tank) from the control group or the experimental group. For the experimental group, the crabs which had typical paroxysmal tremors of the pereiopod were randomly collected to prepare thoracic ganglion samples. After wiping with 75% alcohol, thoracic ganglions from the crabs were sampled and washed with ice-cold PBS three times to remove hemocytes and other tissue cells. Samples were immediately frozen in liquid nitrogen and stored at −80 °C until protein extraction. All experiments were repeated three times.

### Transmission Electron Microscopy and Quantification of S. eriocheiris

To ensure whether *S. eriocheiris* invaded the thoracic ganglion of the crab at 9 days after *S. eriocheiris* infection (the crabs with typical paroxysmal tremors of the pereiopod), transmission electron microscopy (TEM) was carried out as described by Wang *et al*. (13) and observed with a Hitachi H-7650 TEM (Japan).

Every three crabs in the *S. eriocheiris*-infection group were collected for analysis of *S. eriocheiris* copies at 0, 1, 3, 5, 7, and 9 days. The number of *S. eriocheiris* copies in the crab thoracic ganglions was determined by absolute real-time PCR as described by Ding *et al*. (16) and using the primers Se-QF and Se-QR (supplemental Table S1). All samples were run three times.

### TMT-Based Quantitative Phosphoproteomic Analysis of Crab Thoracic Ganglions

#### Experimental Design

For phosphoproteomic analysis, the thoracic ganglion samples of equal mass were collected from control group (R2 medium-injected crabs, DZ) and experiment group (*S. eriocheiris*-infected crabs exhibiting characteristic pereiopod tremors, SY) at 9 days after injection for protein extract and phosphoproteomic analysis, respectively. The entire experiment was independently repeated three times. Detailed information regarding all materials, reagents, and instrumentation used is provided in supplemental Table S2.

#### Protein Extraction, Trypsin Digestion, and TMT Labeling

The sample was sonicated three times on ice using a high-intensity ultrasonic processor (Scientz) in lysis buffer (8 M urea, 2 mM EDTA, 10 mM DTT, and 2% phosphatase inhibitor cocktail V). The protein concentration was determined with a 2-D Quant kit (GE Healthcare Bioscience, 80-6483-56) according to the manufacturer’s instructions. For trypsin digestion, the protein sample was diluted by adding 100 mM TEAB to a urea concentration of less than 2 M. Finally, trypsin was added at a 1:50 trypsin-to-protein mass ratio for the first digestion overnight and a 1:100 trypsin-to-protein mass ratio for a second 4 h digestion. After trypsin digestion, the peptide was desalted by a Strata X C18 SPE column (Phenomenex) and vacuum-dried. Tryptic peptides were first dissolved in 0.5 M TEAB. Each channel of peptide was labeled with their respective TMT reagent (based on manufacturer’s protocol, Thermo Scientific, 90061) and incubated for 2 h at room temperature. Five microliters of each sample were pooled, desalted, and analyzed by MS to check labeling efficiency. After labeling efficiency check, samples were quenched by adding 5% hydroxylamine. The pooled samples were then desalted with Strata X SPE column (Phenomenex) and dried by vacuum centrifugation.

#### Affinity Enrichment and LC-MS/MS Analysis

The sample was then fractionated into fractions by high pH reverse-phase HPLC using an Agilent 300Extend-C18 column (5 μm particles, 4.6 mm ID, 250 mm length). Briefly, peptides were separated with a gradient of 2% to 60% acetonitrile in 10 mM ammonium bicarbonate, pH 10, over 80 min into 80 fractions. Then, the peptides were combined into 18 fractions and dried by vacuum centrifuging. Peptide mixtures were first incubated with immobilized metal ions affinity chromatography microspheres suspension with vibration in loading buffer (50% acetonitrile/0.5% acetic acid). To remove the nonspecifically adsorbed peptides, the immobilized metal ions affinity chromatography microspheres were washed with 50% acetonitrile/0.5% acetic acid and 30% acetonitrile/0.1% trifluoroacetic acid, sequentially. To elute the enriched phosphopeptides, the elution buffer containing 10% NH4OH was added, and the enriched phosphopeptides were eluted with vibration. The supernatant containing phosphopeptides was collected and lyophilized for LC-MS/MS analysis. Briefly, the tryptic peptides were dissolved in solvent A (0.1% formic acid (FA), 2% acetonitrile in water), directly loaded onto a reversed-phase precolumn (Acclaim PepMap 100, Thermo). The mobile phase consisted of solvent A and solvent B (0.1% FA and 98% acetonitrile/in water). Peptides were separated with the following gradient: 0 to 50 min, 4% to 22%B; 50 to 62 min, 22% to 35%B; 62 to 66 min, 35% to 85%B; and 66 to 70 min, 85% and all at a constant flow rate of 300 nl/min on a EASY-nLC 1000 UPLC system (Thermo). The peptides were subjected to NSI source followed by tandem mass spectrometry (MS/MS) in Q ExactiveTM Plus (Thermo) coupled online to the UPLC. Intact peptides were detected in the Orbitrap at a resolution of 70,000. Peptides were selected for MS/MS using NCE setting as 28; ion fragments were detected in the Orbitrap at a resolution of 17,500. A data-dependent procedure that alternated between 1 MS scan followed by 20 MS/MS scans was applied for the top 20 precursor ions above a threshold ion count of 5.0E3 in the MS survey scan with 15.0s dynamic exclusion. The electrospray voltage applied was 2.0 kV. Automatic gain control was used to prevent overfilling of the orbitrap; 5E4 ions were accumulated for generation of MS/MS spectra. For MS scans, the m/z scan range was 350 to 1800. Fixed first mass was set as 100 m/z.

#### Database Search

The resulting MS/MS data were processed using MaxQuant with an integrated Andromeda search engine (v.1.4.1.2). Tandem mass spectra were searched against the *E. sinensis* transcriptome database (including 39,636 proteins) (17) and *S. eriocheiris* genome (including 1242 proteins) (18) concatenated with the reverse decoy database. Trypsin/P was specified as cleavage enzyme allowing up to two missing cleavages, five modifications per peptide, and five charges. Mass error was set to 10 ppm for precursor ions and 0.02 Da for fragment ions. Carbamidomethylation on Cys was specified as fixed modification and oxidation on Met, phosphorylation on Ser, Thr, and Tyr, and acetylation on protein N-terminal were specified as variable modifications. False discovery rate thresholds for protein, peptide, and modification sites were specified at 1%. The minimum peptide length was set at 7. For the quantification method, TMT-6 plex was selected. All the other parameters in MaxQuant were set to default values. The site localization probability was set as > 0.5.

#### Quantitative and Differential Analysis

For quantitative analysis, the raw LC-MS datasets were first searched against database and converted into matrices containing reporter intensity of peptides across samples. The relative quantitative value of each modified peptide with localization probability >0.5 was then calculated based on the intensity information by the following steps: First, the intensities of modified peptides *(I)* were centralized and transformed into relative quantitative values *(U)* of modified peptides in each sample. The formula is listed as follow: *Uij* = *Iij/Mean(Ij)* (i denotes the sample and j denotes the modified peptide). To adjust the systematic bias of the identified modified peptides amount among different samples in the process of mass spectrometry detection, the relative quantitative value of modified peptides needs to be corrected by median value as follows: *Rij* = *Uij/Median(Ui)*. To calculate the fold change between experiment samples (SY) and control samples (DZ), the formula is listed as following: *FC*_*SY/DZ,k*_ = *R*_*SYk*_*/R*_*DZk*_, where R denotes the relative quantitative value of the site and k denotes the modification site. We only used ratios with *p value*s < 0.05, and only fold changes >1.5 were considered significant.

#### Bioinformatics Analysis

Gene ontology (GO), Kyoto Encyclopedia of Genes and Genomes (KEGG), and protein domain analyses were used to analyze the identified differentially expressed proteins. For each category, a two-tailed Fisher’s exact test was employed to test the enrichment of the differentially expressed proteins against all identified proteins. Correction for multiple hypothesis testing was carried out using standard false discovery rate control methods. A corrected *p value* < 0.05 was considered significant.

### Phosphorylation Peptide Synthesis, Antibody Preparation, Immunohistochemistry, and Western Blot Analysis

To validate the results of the differentially expressed phosphorylated proteins identified in this article, 12 significantly differentially expressed phosphorylated peptides (Table 1) were selected for artificial synthesis. After coupling with immunogenicity enhancement factors, synthetic phosphorylated peptides were used to immunize New Zealand White rabbits seven times at 2-weeks intervals to prepare antibodies. Rabbit serum was collected at 7 days after the last immunization. The titer and specificity of antibodies were determined by ELISA and Western blot, respectively. Unfortunately, only four phosphorylation peptide antibodies (GSK3*β*-Ser9, VAMP-Ser72, SYN-Ser134, and SNAP25-Ser102) and *β*-catenin antibody met the requirements for subsequent experiments.

**Table 1:** The phosphorylated peptides mentioned and identified in this paper

Protein names	Modified sequence	Position	Fold
**Microtubule-associated protein tau(tau)**^a^	_SSDPS(ph)PMK_	77	1.81
**Microtubule-associated protein tau(tau)**	**_ASSEANLS(ph)R_**	**284**	**1.68**
Map/microtubule affinity-regulating kinase (MARP)	_TPVS(ph)PVSTNR_	597	1.77
P38	_PTESEMT(ph)GY(ph)VATR_	214/216	1.96/1.58
Tau-tubulin kinase 1 (TTBK)	_S(ph)CEDLLEEGSEESSK_	1087	1.58
**cAMP-dependent protein kinase catalytic subunit (PKA)**	**_AVVHSAS(ph)LQR_**	**139**	**0.57**
Calcium/calmodulin-dependent protein kinase type II (CAMKII)	_VAS(ph)VVHRQET(ph)VDCLR_	279	0.64
**Calcium/calmodulin-dependent protein kinase type II (CAMKII)**	**_SRS(ph)IITK_**	**319**	**0.27**
Calcium/calmodulin-dependent protein kinase type II (CAMKII)	_KGDGS(ph)QVK_	328	0.54
Calcium/calmodulin-dependent protein kinase type II (CAMKII)	_SQVDRS(ph)T(ph)T(ph)VIAR_	356/357/	0.52/0.52/
Calcium/calmodulin-dependent protein kinase type II (CAMKII)	_ATEQLIDCINS(ph)GDFEGYTK_	358	0.36
***Glycogen synthase kinase-3 beta(GSK-3β)***^b^	***_TTS(ph)FADGNK_***	***9***	***0.38***
Segment polarity protein dishevelled homolog DVL-3	_LQPAMS(ph)R_	230	0.52
Inositol 1,4,5-trisphosphate receptor (IP3R)	_DASSTS(ph)LSGR_	482	0.6
**Protein kinase C (PKC)**	**_TELT(ph)PTDK_**	**82**	**0.5**
Calmodulin	_HVMT(ph)NLGEK_	113	0.44
***Vesicle-associated membrane protein(VAMP)***	***_DQKLS(ph)ELDDR_***	***72***	***0.58***
**Vesicle-associated membrane protein(VAMP)**	**_ADALQQGAS(ph)QFEQQAGK_**	**86**	**0.66**
**Synaptosome-associated protein of 25 kda(SNAP25)**	**_SNEVTDDTLES(ph)TRR_**	**33**	**0.62**
Synaptosome-associated protein of 25 kda(SNAP25)	_MLS(ph)LCEESK_	39	0.39
***Synaptosome-associated protein of 25kda(SNAP25)***	***_TSS(ph)QFKEDESTWK_***	***102***	***0.65***
***Syntaxin-12 (SYN)***	***_ADS(ph)DAQFFEDSR_***	***134***	***0.66***
**Synaptotagmin(SYT)**	**_SPTGNKS(ph)PPGAPR_**	**104**	**0.35**
V-atpase	_SRT(ph)WDFQPDR_	137	0.64

a Font bold was the phosphorylated peptides that were selected for antibody preparation.

b Italics bold was the phosphorylated peptides were antibody meets subsequent experiments requirements.

The GSK3*β* antibody was prepared by *E. sinensis* GSK3*β* recombinant protein expression in *Eriocheir coli* and immunized rabbits. Antibodies GSK3*β*-pTyr216 (ab68476) and the reference gene protein actin (ab197345) were purchased from Abcam.

To validate the results of differentially expressed phosphorylated proteins, the samples used in immunohistochemistry were the same as those used in the proteomics analysis. The specimens were fixed in 4% (w/v) paraformaldehyde and subsequently embedded in paraffin. Antigen retrieval was performed using 10 mM sodium citrate buffer (pH 6.0). Tissue sections were treated with 3% hydrogen peroxide (at room temperature for 15 min) to prevent endogenous peroxidase activity. For the immunohistochemistry assay, the tissue sections were blocked with 3% bovine serum albumin (BSA) at room temperature for 2 h and then incubated with primary antibody (diluted with PBS appropriately) overnight at 4 °C. HRP-labeled secondary antibody was added and incubated for 1 h at room temperature. Freshly prepared diaminobenzidine chromogenic reagent was added to the marked tissue. The reaction time was managed by observation under a microscope until the nucleus appeared brown–yellow. Then, the developing reaction was stopped by washing under running tap water. Immunoreactive regions were visualized using microscopy.

Western blotting was used to detect the protein expression patterns after *S. eriocheiris* infection following a method described in a previous research paper (12). The protein samples used for Western blot analysis were same as the phosphorylated proteomics analysis. The expression of actin was used as a control. All Western blot analyses were repeated on several occasions, and the gray value of the bands was detected with Image J software.

### Neurotransmitter Analysis of Crab Thoracic Ganglions

Thirty milligrams of thoracic ganglion tissue from each crab from 10 experimental samples and 10 control samples were prepared to extract neurotransmitters. Samples were treated with 200 μl of 0.1% FA in cold water and homogenized. After 800 μl of 0.1% FA in methanol/acetonitrile (1:1, v/v) was added, the samples were vortexed, sonicated for 30 min, and placed at −20 °C for 1 h. After centrifugation at 14,000 rpm at 4 °C for 20 min, the supernatant was collected and vacuum dried.

LC-MS/MS analysis was performed using an Agilent 6410 triple stage quadrupole mass spectrometer equipped with an ESI ion source and an Agilent 1290 HPLC system with an autosampler (Agilent). Briefly, the analysis was separated on an ACQUITY UPLC BEH Amide column (1.7 μm, 2.1 mm × 100 mm, Waters) at 45 °C. The mobile phase, consisting of 0.1% FA in water and acetonitrile, was used with a gradient elution of 0 to 4 min, 2% B; 6 min, 80% B; and 8 to 10 min, 90% B at a flow rate of 0.1 ml/min. ESI-MS/MS conditions were set as follows: gas temperature 450 °C, gas flow 10 min, capillary 4500 V, and nebulizer pressure 45 psi. MS acquisition of 5-hydroxy-L-tryptophan, DOPA, glutamine, acetylcholine, γ-amino-butyric acid, histamine, 3-methoxytyramine, glutamate, and serotonin was performed in electrospray positive ionization multiple reaction monitoring.

### Cloning, Recombinant Expression, and Antibody Preparation of the Main Components of the Wnt Pathway

Total RNA from crabs was extracted using TRIzol reagent following the manufacturer's instructions. RNA quality was assessed by electrophoresis on a 1.2% agarose gel, and the RNA concentration was measured by absorbance at 260 nm on a spectrophotometer. A SMARTer RACE cDNA Amplification Kit (Takara) was used for rapid amplification of cDNA ends (RACE). Two pairs of gene-specific primers were designed based on the corresponding EST sequences (Es*β*-catenin-5′R1, Es*β*-catenin-5′R2, Es*β*-catenin-3′R1, and Es*β*-catenin-3′R2). For 5′-RACE, PCRs were performed with Es*β*-catenin-5′R1 or Es*β*-catenin-5′R2 and Universal Primer A Mix. For 3′-RACE, PCR was performed with Es*β*-catenin-3′R1 or Es*β*-catenin-3′R2 and Universal Primer A Mix. The PCR fragments were cloned into a pMD19-T vector and sequenced by Springen Biotechnology Company (Nanjing).

Homology analysis of nucleotide and amino acid sequences was conducted with BLAST programs (http://blast.ncbi.nlm.nih.gov/Blast.cgi). The deduced amino acid sequence was analyzed with the Expert Protein Analysis System (http://www.expasy.org/). The SignalP 4.1 program was utilized to predict the signal peptide (http://www.cbs.dtu.dk/services/SignalP/). Domain prediction was conducted with the Simple Modular Architecture Research Tool (http://smart.embl-heidelberg.de/). Multiple sequence alignment was performed using ClustalW2 (http://www.ebi.ac.uk/Tools/msa/clustalw2/). The phylogenetic tree was constructed based on the amino sequence alignment by the neighbor-joining algorithm embedded in the MEGA 6 program.

Es*β*-catenin-ORF-F, Es*β*-catenin-ORF-R and EsGSK3β-ORF-F, and EsGSK3β-ORF-R were designed to amplify the open reading frames (ORFs) of the Es*β*-catenin and EsGSK3β genes. The product was ligated into the pET-30a (+) expression vector. *E. coli* BL21 (DE3) was transformed with the resulting recombinant plasmids pET-30a-Es*β*-catenin and pET-30a-EsGSK3β to induce recombinant expression using isopropyl β-D-1-thiogalactopyranoside (final concentration 0.5 mM). The temperature for induction of protein production was 37 °C. The recombinant proteins were analyzed by SDS–PAGE. High-affinity nickel-nitrilotriacetic acid resin (Jinsite) was used to purify the recombinant proteins according to the manufacturer's instructions. The antibody was prepared as described above.

### Expression Analysis of Esβ-Catenin and EsGSK3β

The mRNA transcriptions of Es*β*-catenin and EsGSK3β in different tissues, including hemocytes, heart, hepatopancreas, gill, intestine, nerve, and muscle of untreated crabs, were determined by quantitative real-time reverse transcription PCR (qRT–PCR) using the primers Es*β*-catenin-qR, Es*β*-catenin-qR, EsGSK3β-qF, and EsGSK3β-qR. The temporal transcription of Es*β*-catenin and EsGSK3β in hemocytes was determined after challenge with *S. eriocheiris*. The crabs were randomly divided into two groups. One hundred crabs were injected individually with 100 μl live *S. eriocheiris* (10^8^ cells/ml) as an experimental group. For the control group, 100 crabs were injected with 100 μl R2 medium. Every five individuals were randomly sampled at 0, 1, 3, 5, 7, and 9 days postchallenge. Hemocytes were collected to subsequently extract total RNA and protein. Total protein, cytoplasmic protein, and nuclear protein extracts were obtained according to the manufacturer’s protocol or protein extraction kits (Beyotime). qRT–PCR and Western blotting were used to determine gene expression levels at the mRNA or protein level.

Hemocytes at 0 and 9 days postchallenge were fixed with 4% paraformaldehyde, deposited onto a glass slide, washed with PBS (140 mM NaCl and 10 mM sodium phosphate, pH 7.4) and incubated in 0.2% Triton X-100 at 37 °C for 5 min. After washing with PBS, the hemocytes on the glass slides were blocked with 3% BSA (30 min, 37 °C) and incubated with anti-Es*β-*catenin, anti-EsGSK3β, anti-EsGSK3β-Ser9, and anti-EsGSK3β-Tyr214 (1:200 in 3% BSA) overnight at 4 °C. After washing with PBS and incubating with 3% BSA for 10 min, the hemocytes were incubated with Alexa Fluor 647-labeled secondary antibody (Abcam, ab150079; 1:1000 ratios, diluted in 3% BSA) at 37 °C for 1 h in the dark. The hemocytes were incubated with 4′-6-diamidino-2-phenylindole dihydrochloride (1 μg/ml in PBS) for 10 min at room temperature and washed six times. Fluorescence was observed using a fluorescence microscope (Nikon A1R).

### RNA Interference of Esβ-Catenin and EsGSK3β and Their Effect on S. eriocheiris Infection

The double-stranded RNA (dsRNA) of Es*β*-catenin, EsGSK3β, and GFP was synthesized separately using the manufacturer’s instructions from an *in vitro* transcription T7 kit (Takara) using the primer pairs Es*β*-catenin-RNAiF/Es*β*-catenin-RNAiR and EsGSK3β-RNAiF/EsGSK3β-RNAiR. Agarose gel electrophoresis and spectrophotometry were utilized to monitor the synthesis of dsRNAs. For RNA interference, the experimental groups (100 crabs in each group) received 20 μg dsRNA per crab of Es*β*-catenin and EsGSK3β intramuscularly injected, while the control groups were injected with equivalent GFP dsRNA and PBS, respectively. The injection was repeated after 24 h. qRT–PCR (primer pairs Es*β*-catenin-qiF/Es*β*-catenin-qiR and EsGSK3β-qiF/EsGSK3β-qiR), Western blot, and immunofluorescence assays were performed to measure the RNA interference efficiency at 48 h, 72 h, and 96 h post-dsRNA injection. Actin was used as an internal control. For the EsGSK3β RNA interference group, Western blotting was also used to analyze the effect of EsGSK3β knockdown on Es*β*-catenin accumulation in the nucleus and cytoplasm. To further analyze the effect of Es*β*-catenin and EsGSK3β RNA interference, qRT–PCR was used to analyze the transcription of Es*β*-catenin target genes Axin-1, transcription factor AP-1 (AP-1), matrix metalloproteinase 2 (MMP2), and MMP14 at 48 h, 72 h, and 96 h post-dsRNA injection.

Forty-eight hours after Es*β*-catenin RNA interference, the crabs received the third injection, 100 μl of *S. eriocheiris* (10^8^ cells/ml), or were mock-challenged with PBS (100 μl) as a control. The crabs were cultured in tanks for approximately 2 weeks following infection. Hemocytes of five random crabs were sampled from each group at 0, 1, 3, 5, 7, and 9 days postchallenge and used to measure the copes of *S. eriocheiris*. The cumulative mortality of each group was recorded every 24 h. Differences were analyzed by using the Kaplan–Meier plot (log-rank c2 test) method with GraphPad Prism software.

### Primary Hemocytes of Crabs Treated With a Wnt Pathway Activator (SB-216763) and Its Effect on S. eriocheiris Infection

The crabs were immersed in distilled water for 20 min and surface-sterilized with 75% ethanol, paying particular attention to the pereiopods and thorax. A syringe was used to collect 1 ml hemolymph by puncturing the thorax at the base of a pereiopod. The hemolymph was immediately expelled into 1 ml of sterile anticoagulant solution ACD-B in a 10 ml tube in a Class II Biological Safety Cabinet (ESCO). The hemolymph was centrifuged at 2000*g* for 4 min at 4 °C to collect the cells. The cells were resuspended gently in 1 ml culture medium, seeded into 1 ml of culture medium in the wells of a 6-well tissue culture plate (Corning), and incubated at 28 °C.

SB-216763 is an ATP-competitive inhibitor of GSK3β that specifically downregulates the activity of GSK3β by decreasing the phosphorylation level of GSK3β-Tyr214 (19, 20) to activate the Wnt-*β*-catenin signaling pathway, which ultimately increased *β*-catenin expression. To further study the role of the Wnt pathway in the process of *S. eriocheiris* invading crab hemocytes SB-216763 was used to activate the Wnt signaling pathway. Briefly, based on previous studies, different concentrations of SB-216763 (CST, #13621; 0, 1, 5, and 10 μM) were used. Two hours later, Western blot and confocal experiments were used to select the optimum concentration for hemocytes.

Primary hemocytes of crabs were subsequently treated with the optimum concentration (5 μM) of SB-216763 as the experimental group. The control group was treated with the equivalent amount of DMSO. After 2 h of treatment with SB-216763 or DMSO, the hemocytes were infected with 100 μl *S. eriocheiris* (the ratio of spiroplasma to cells was 30:1). At 0, 12, and 24 h post-*S. eriocheiris* infection, the *S. eriocheiris* copies in hemocytes were quantified by qRT–PCR, and the viability of hemocytes was determined by an EnoGene Cell Counting Kit-8 (CCK-8) (Beyotime) according to the manufacturer’s instructions.

### Coimmunoprecipitation Analysis of the Interaction Between Esβ-Catenin and EsGSK3β

*Drosophila* S2 cells were cotransfected with pAc5.1-Es*β*-catenin-V5 and pAc5.1-EsGSK3*β*-GFP at a ratio of 1:1 for the control group cotransfected with pAc5.1-*Esβ*-catenin-V5 and pAc5.1-GFP. At 48 h posttransfection, cells were harvested and lysed on ice with IP lysis buffer following the manufacture’s manual (Beyotime). The samples were incubated with 1 μg of tag antibody (anti-GFP or anti-V5) overnight at 4 °C. Protein A/G beads (Transgen) were added into the samples and incubated at 4 °C under rotation for 2 h. Then, the beads were collected by centrifugation (1000*g* for 5 min) and washed three times with IP lysis buffer. The resulting pellets were resuspended in 6 × SDS loading buffer and boiled to elute proteins from beads. Both cell lysates and bead-bound proteins were analyzed by Western blot using tag antibody (anti-GFP or anti-V5).

### EsGSK3β Key Amino Acid Site Tyr214 Affect the Esβ-Catenin Transferred From the Cytoplasm to the Nucleus

Bioinformatics analysis showed that the 214th amino acid Tyr of EsGSK3β may regulate the protein activity phosphorylation. To further explore the effect of EsGSK3β Tyr214 phosphorylation on the Esβ-catenin nuclear translocation, we mutated the EsGSK3β 214th Tyr as Asp (D) to simulate continuous phosphorylation at this site and then cotransfected pAc5.1-Es*β*-catenin-GFP with pAc5.1-EsGSK3*β*-GFP, pAc5.1-EsGSK3*β*/214D-GFP, and pAc5.1-GFP, respectively. At 48 h post-transfection, cells were harvested, and cytoplasmic protein and nuclear protein were extracted following the manufacture’s manual (Beyotime). Western blot was used to analyze the samples using anti-GFP antibody.

### Esβ-Catenin and EsGSK3β Key Amino Acid Site Mutation and the Effect on *S. eriocheiris*-Infected S2 Cell

To further explore the effect of Esβ-catenin and EsGSK3β key amino acid site mutation on *S. eriocheiris-*infected S2 cell, the Esβ-catenin key amino acid Ser44, Ser48, and Thr52 were mutated as Ala (A) to simulate continuous dephosphorylation or mutated as Asp (D) to simulate continuous phosphorylation and then transfected the pAc5.1-EsGSK3β-GFP, pAc5.1-EsGSK3β/214D-GFP, pAc5.1-Esβ-catenin, pAc5.1-Esβ-catenin44/48/52A, pAc5.1-Esβ-catenin44/48/52D, and pAc5.1-GFP to S2 cell, respectively. After 24 h post-transfection, transfected cells were infected with *S. eriocheiris* at an MOI of 30 for 48 h, and the viability of hemocytes was determined by CCK-8 (Beyotime, China) according to the manufacturer’s instructions. At the same time, qRT–PCR and flow cytometry were used to analyze the *S. eriocheiris* copies and apoptotic rate of S2 cells which were transfected with pAc5.1-Esβ-catenin44/48/52A, pAc5.1-Esβ-catenin44/48/52D, pAc5.1-Esβ-catenin, and pAc5.1-GFP, respectively.

### Transcriptomic Analysis of the Effect After Esβ-Catenin and EsGSK3β RNA Interference

To determine how Wnt-*β*-catenin regulates the innate immune response in crab hemocytes, Es*β*-catenin and EsGSK3β RNA interference *de novo* transcriptomic analysis was performed to identify the innate immune–related genes regulated by this pathway. The methods for carrying out Es*β*-catenin and EsGSK3β RNA interference were the same as described above. After dsRNA (dsRNA of Es*β*-catenin, EsGSK3β, and GFP) injection for 72 h, the hemocytes of the crabs were collected and used for transcriptome sequencing analysis. All experiments were repeated three times. Using a fold change ≥2 and *p value* ≤ 0.05 in gene transcription as a benchmark for physiologically significant change, qRT–PCR was used to verify the transcription of representative differentially expressed innate immune–related genes, including lipopolysaccharide-β-1,3-glucan binding protein (LGBP), serine protease (SP), spaetzle, anti-lipopolysaccharide factor 1 (ALF1), ALF2, ALF3, crustin 1, and hyastatin.

## Results

### Transmission Electron Microscopy and Quantitative S. eriocheiris

**Fig. 1:**
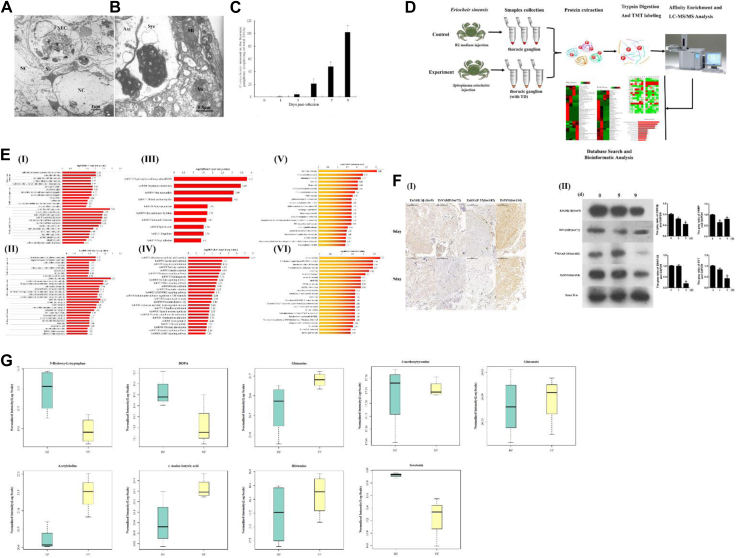
***S. eriocheiris* infection restrained crab thoracic ganglion Wnt pathway and disordered the neurotransmitter metabolism.***A* and *B*, TEM of the thoracic ganglion after crabs were infected with *S. eriocheiris*. (*A*) *S. eriocheiris* in the nerve tissue; (*B*) *S. eriocheiris* in the motor end-plate. *C*, *S. eriocheiris* copies in the crab thoracic ganglion throughout the infection. *D*, the workflow of phosphoproteomics. *E*, GO-based enrichment analysis of upregulated (Ⅰ) and downregulated (Ⅱ) phosphorylated proteins; KEGG pathway-based enrichment analysis of upregulated (Ⅲ) and downregulated (Ⅳ) proteins; protein domain enrichment analysis of upregulated (Ⅴ) and downregulated (Ⅵ) proteins. *F*, verification of the differential expression of phosphorylated proteins (EsGSK3β-Ser9, EsVAMP-Ser72, EsSYN-Ser134, and EsSNAP25-Ser102) in the thoracic ganglion of crabs after *S. eriocheiris* infection using immunohistochemistry (I) and Western blotting (II). *G*, the neurotransmitter in the thoracic ganglion of crabs after *S. eriocheiris* infection. Statistical significance is indicated (∗, *p* < 0.05). The data are the means ± SD for 10 independent experiments. Axt, axon terminal; GO, Gene ontology; KEGG, Kyoto Encyclopedia of Genes and Genomes; NC, nerve cell; NEC, neurogliocyte; Mi, mitochondria; S, *S. eriocheiris*; Syc, synaptic cleft; TEM, transmission electron microscopy.

TEM results showed that when the crab had typical paroxysmal tremors of the pereiopod, many *S. eriocheiris* were found in the neuroglial cell (Nec) and motor endplate (the connections between nerve and muscle cells) of the crab (Fig. 1, *A* and *B*). The nervous tissue cells of the diseased crab had obvious pathological changes, and the tissue structure was destroyed. The neuroglial cell and connective tissue cell contain the *S. eriocheiris* inclusion body, and *S. eriocheiris* reproduces in the inclusion body and causes host cell enlargement, swelling, and disintegration or rupture. This result directly demonstrated that nervous tissue was the target tissue of *S. eriocheiris*. The copies of *S. eriocheiris* in the crab thoracic ganglion were measured through absolute real-time PCR (Fig. 1*C*). In the thoracic ganglions, the copy number of the *S. eriocheiris* group was markedly increased from 3 days to 9 days. *S. eriocheiris* copies reached a maximum of approximately 1.0 × 10^2.03^ copies/ng DNA at 9 days.

### TMT Quantification Phosphoproteomic Analysis

To explore the pathways or proteins participating in the occurrence of crab TD, quantitative phosphoproteome analysis was used to obtain the differentially expressed proteins in the thoracic ganglion between the healthy and the diseased crab (9 days after *S. eriocheiris* infection and the crab with pereiopods, typically paroxysmal tremors) through TMT labeling and affinity enrichment followed by high-resolution LC-MS/MS analysis, and the workflow of phosphoproteomics was shown in Figure 1*D*. Altogether, 6040 phosphorylation sites in 2451 protein groups were identified, among which 4157 sites in 1862 proteins were quantified against the *E. sinensis* transcriptome database. Soft motif-x was used to analyze the identified phosphorylation peptides in this study, and all the database protein sequences were used as background database (supplemental Fig. S1*A*, supplemental Table S3). Proteins with quantitative ratios above 1.5 or below 1/1.5 were deemed significant. Among the quantified proteins, 349 phosphorylated proteins were upregulated, and 331 phosphorylated proteins were downregulated when compared to the control sample (supplemental Table S4) such as synaptotagmins (SYTs), synaptosome-associated protein of 25 kDa (SNAP-25), Tau-tubulin kinase 1, synapsin (SYN), nitric oxide synthase, calcium/calmodulin-dependent protein kinase type II (CaMKII), vesicle-associated membrane protein (VAMP), glycogen synthase kinase-3 beta, and so on. The subcellular location of upregulated (supplemental Fig. S1*B*I and supplemental Table S4) and downregulated (supplemental Fig. S1*B*II and supplemental Table S4) proteins identified in this article were analyzed. Against the *S. eriocheiris* genomic total, 14 phosphorylation sites and 13 phosphorylated peptides in 11 proteins were identified (supplemental Fig. S2 and supplemental Table S5).

To further understand the function and feature of the identified and quantified proteins, all the identified different expression proteins phosphoproteins were used for GO, KEGG, and domain annotation and enrichment analysis, and the *E. sinensis* thoracic ganglion transcriptome was set as the background dataset. For GO annotation and enrichment, the distribution bar charts of upregulated and downregulated phosphoproteins for molecular function, cellular component, and biological process are shown in Figure 1*E*Ⅰ and Ⅱ, respectively. For the upregulated phosphorylated protein, from the molecular function perspective, calmodulin-related proteins were significantly enriched. For cellular component, cytoskeletal-related and associated with muscle movement were also significantly enriched. From the biological process perspective, there were lots of cytoskeleton regulation, and phosphorylation-related process was significantly enriched. For the downregulated phosphorylated protein, there were also many proteins or biological process related to the composition of nervous system and nervous system development, and transmission of nerve signals were significantly enriched. Based on cellular component synapse, synapse part and voltage-gated sodium channel complex were enriched. Calcium ion binding, cation channel activity, and cation transmembrane transporter activity were enriched according to molecular function. At the same time, many processes were significantly enriched according to biological process, such as sodium ion transport, cation transport, Wnt signaling pathway, secretion, and so on.

For KEGG enrichment analysis, the pathways with a Fisher's exact test *p value* < 0.05 were significantly highlighted. The results showed that in the upregulated phosphoproteins (Fig. 1*E*Ⅲ, supplemental Table S6), hypertrophic cardiomyopathy (ko05410), tryptophan metabolism (ko00380), viral myocarditis (ko05416), and one carbon pool by folate (ko00670) were the main highlighted pathways. For downregulated phosphoproteins (Fig. 1*E*Ⅳ, supplemental Table S6), there were more pathways significantly enriched than upregulated phosphoproteins. It is worth noting that there were many pathways relevant to normal regulation of nervous system, such as Wnt signaling pathway (ko04310), calcium signaling pathway (ko04020), dopaminergic synapse (ko04728), synaptic vesicle cycle (ko04721), cGMP-PKG signaling pathway (ko04022), and vascular smooth muscle contraction (ko04270). The protein domain enrichment analysis of upregulated proteins (Fig. 1*E*Ⅴ, supplemental Table S7) and downregulated proteins (Fig. 1*E*Ⅵ, supplemental Table S7) was carried out. Consistent with the above results, many nervous system development and nerve signal transmission domains were also enriched, such as LIM-type, myosin tail, EGF-like calcium-binding domain, and neurofascin/L1/NrCAM C-terminal domain. These results demonstrated that when *S. eriocheiris* infected the thoracic ganglion of the crabs, this pathogen influenced these proteins or pathways to destroy the normal regulation of the nervous system.

### Verification of the Significantly Expressed Phosphorylated Proteins by Immunohistochemical and Western Blot Analysis

To validate the results of TMT, four phosphorylated peptides (GSK3*β*-Ser9, VAMP-Ser72, SNAP25-Ser102, and SYN-Ser134) were selected for chemical synthesis and antibody preparation. Immunohistochemistry was used to detect the expression of these four proteins in the crab thoracic ganglion. The results showed that the phosphorylation sites of the four proteins were all significantly downregulated after *S. eriocheiris* infection. These changing trends of the target sites were similar to the results obtained by phosphorylated proteomics (Fig. 1*F*Ⅰ). The Western blot results were also similar to the phosphorylated proteomics and immunohistochemical results (Fig. 1*F*Ⅱ).

### Neurotransmitter Analysis in the Crab Thoracic Ganglion

The levels of the relevant neurotransmitter metabolites 5-hydroxy-L-tryptophan, DOPA, glutamine, acetylcholine, γ-amino-butyric acid, histamine, 3-methoxytyramine, and glutamate were measured using LC-MS/MS (Fig. 1*G*). 5-hydroxy-L-tryptophan and serotonin were significantly decreased in the *S. eriocheiris* infection group (crab with typical paroxysmal tremors of the pereiopod) compared with the control group. Acetylcholine and γ-amino-butyric acid were increased in the experimental group.

### Molecular Characterization of Esβ-Catenin and EsGSK3β in the Wnt Signaling Pathway

The full-length cDNA sequence of Es*β*-catenin was obtained by the EST sequence from transcriptome data combined with 5′ and 3′-RACE PCR amplification. The Es*β*-catenin cDNA sequence was 3061 bp in length and comprised a 143 bp 5′-untranslated region, a 467 bp 3′-untranslated region containing a poly (A) tail, and a 2451 bp ORF that encoded a protein of 816 amino acids. The deduced Es*β*-catenin was predicted to have a molecular mass of 89.39 KDa and a theoretical isoelectric point of 6.17 (supplemental Fig. S2*A*Ⅰ). SMART analysis of the Es*β*-catenin protein contained the typical structure of *β*-catenin, a GSK-3*β* consensus phosphorylation site of 21 amino acids located at the N-terminal region, a 28-aa Coed coil region at 78 to 105 aa, followed by a central region with 12 continuous Armadillo/*β*-catenin-like repeat domains, and a C-terminal region (supplemental Fig. S2*A*Ⅱ). Sequence alignment showed that Es*β*-catenin shared 97.30%, 82.28%, 73.70%, 65.45%, and 65.45% of its identity with those of *L. vannamei*, *Parhyale hawaiensis*, *Drosophila melanogaster*, *Mus musculus*, and *Homo sapiens*, respectively (supplemental Fig. S2*A*Ⅲ). Importantly, the phosphorylation sites of *β*-catenin (Ser33, Ser37, and Thr41) target modified by GSK3β were highly conserved in Es*β*-catenin (respectively correspond Ser44, Ser48, and Thr52). A neighbor-joining phylogenetic tree of Es*β*-catenin protein and its homologs from other species showed Es*β*-catenin was clustered with other crustaceans (supplemental Fig. S2*A*Ⅳ).

The ORF of EsGSK3β was amplified using the sequence from GenBank (GenBank accession no. KT336322). By sequence conservation analysis, we found that EsGSK3β was highly conserved and shared 70.48% with *M. musculus* and *H. sapiens* (supplemental Fig. S2*B*Ⅰ). Importantly, two phosphorylation sites (Ser9 and Tyr214), which play important roles in regulating the activity of this protein, and many key amino acid residues in the catalytic segment (Lys83, Thr136, Gln183, etc.) or activation segments (Arg94, Arg178 and Lys203) were highly conserved in GSK3β from *E. sinensis*, *M. musculus*, and *H. sapiens*. Further SMART analysis showed that the structure of EsGSK3β was highly similar to that of mouse GSK3β (supplemental Fig. S2*B*Ⅱ and Ⅲ).

The recombinant EsGSK3β had an apparent molecular weight of approximately 48 kDa. After denaturation and renaturation, recombinant EsGSK3β was purified by affinity chromatography (supplemental Fig. S2*B*Ⅳ). Rabbit antiserum was also reactive with a constituent that had an apparent molecular weight of 44 kDa, corresponding to the predicted molecular weight (supplemental Fig. S2*B*Ⅴ). Unfortunately, recombinant Es*β*-catenin cannot be successfully expressed in the *E. coli* expression system.

### *S. eriocheiris* Infection Enhances EsGSK3β Phosphorylation Activity to Accelerate Esβ-Catenin Degradation and Restrain the Wnt-β-Catenin Signaling Pathway

**Fig. 2:**
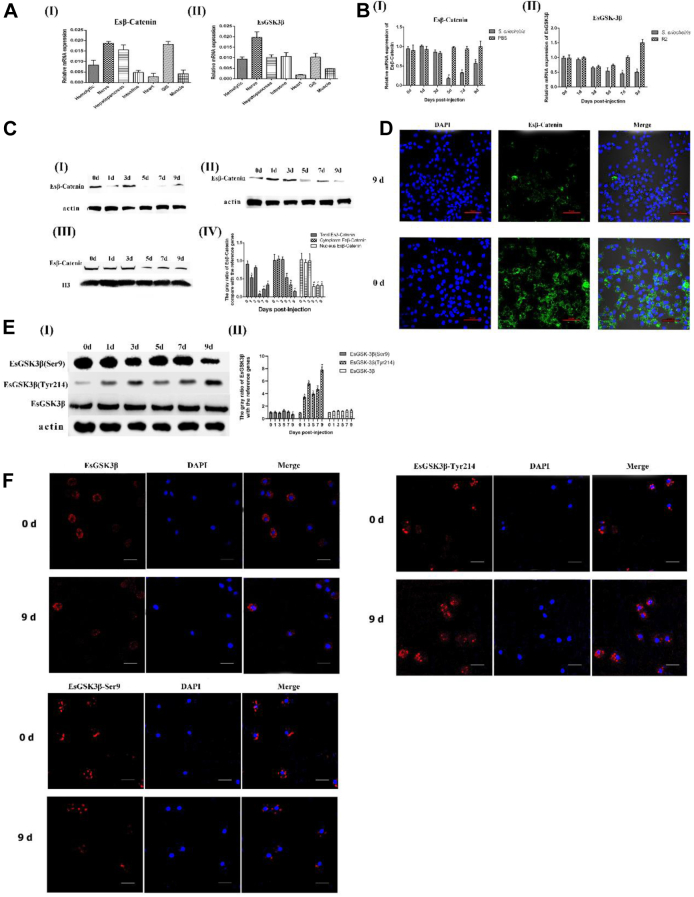
***S. eriocheiris* infection restrained the crab hemocytes Wnt pathway.***A*, tissue distributions of Es*β*-catenin (I) and EsGSK3β (II) in healthy crabs. *B*, temporal transcription profiles of Es*β*-catenin and (Ⅰ) EsGSK3β (Ⅱ) transcripts in crab hemocytes after *S. eriocheiris* infection. *C*, Western blot analysis of the expression levels of Es*β*-catenin protein (Ⅰ: Total Es*β*-catenin; Ⅱ: Cytoplasm Es*β*-catenin; Ⅲ: Nucleus Es*β*-catenin) in hemocytes of *E. sinensis* after *S. eriocheiris* infection; Ⅳ: The corresponding gray value analysis of Western blot; Actin as the reference gene. *D*, immunofluorescence analysis of the expression levels of Es*β*-catenin protein in hemocytes of crabs after *S. eriocheiris* infection on the 0^th^ day and ninth day. *E*, Western blot detection of the expression levels of the total EsGSK3β protein and two forms (inactivity form EsGSK3β-Ser9 and activity form EsGSK3β-Tyr214) in hemocytes of crabs after *S. eriocheiris* infection (I). The corresponding gray value analysis of Western blot; Actin was used as the reference gene (II). *F*, immunofluorescence analysis of the expression levels of the total EsGSK3β protein and two forms (inactivity form EsGSK3β-Ser9 and activity form EsGSK3β-Tyr214) in hemocytes of crabs after *S. eriocheiris* infection on the 0^th^ day and ninth day; Bar: 10 μM. Statistical significance is indicated (∗, *p* < 0.05). The data are the means ± SD for three independent experiments. DAPI, 4′-6-diamidino-2-phenylindole dihydrochloride.

qRT–PCR was employed to quantify Es*β*-catenin and EsGSK3β mRNA transcription in hemolytic, nerve, hepatopancreas, intestine, heart, gill, and muscle tissues. Both Es*β*-catenin and EsGSK3β were transcribed in all tissues of the crab (Fig. 2*A*Ⅰ and Ⅱ). The tissue with the highest transcription of Es*β*-catenin and EsGSK3β was nervous tissue. To verify the function of the Wnt pathway in the immune response of crab, as the first tissue of *S. eriocheiris* infection and most important immune cell, the transcription level of Es*β*-catenin in hemocytes after *S. eriocheiris* infection was detected by qRT–PCR. During *S. eriocheiris* infection, Es*β*-catenin mRNA levels in hemocytes decreased significantly from 5 days to 9 days (*p* < 0.05) (Fig. 2*B*Ⅰ). Similar to Es*β*-catenin, the transcription level of EsGSK3β in hemocytes also decreased significantly from 5 days to 9 days (*p* < 0.05) (Fig. 2*B*Ⅱ). Western blot analysis showed that the protein expression of total, cytoplasmic, and nuclear Es*β*-catenin was significantly downregulated in crab hemocytes after *S. eriocheiris* infection (Fig. 2*C*). Immunocytochemical assay results also showed that the Es*β*-catenin expression level was significantly decreased at 9 days after *S. eriocheiris* infection (Fig. 2*D*). Both Western blot and immunocytochemical assay results showed that the protein expression level of EsGSK3β was downregulated at 9 days after *S. eriocheiris* infection (Fig. 2, *E* and *F*). At the same time, the expression levels of the two forms of EsGSK3β (inactivity form EsGSK3β-Ser9 and activity form EsGSK3β-Tyr214) were quantified against *S. eriocheiris* infection. The results showed that the phosphorylation levels of EsGSK3β-Ser9 and EsGSK3β-Tyr214 were significantly downregulated and upregulated, respectively. This means that during *S. eriocheiris* infection, the activity of EsGSK3β was upregulated, which may accelerate Es*β*-catenin degradation.

**Fig. 3:**
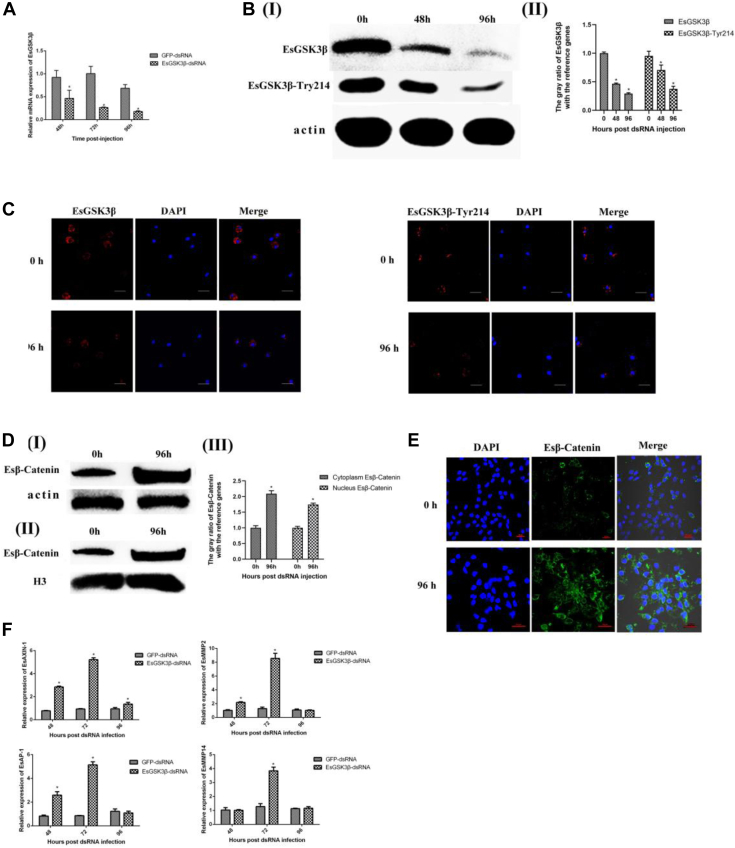
**EsGSK3β negatively regulates Es*β*-catenin stabilization.***A*, *B*, *C*, qRT–PCR (*A*), Western blot (*B*), and immunofluorescence (Bar: 10 μM) (*C*) analysis after EsGSK3β interference. *D*, the effect of EsGSK3β knockdown on Es*β*-catenin expression (Ⅰ: cytoplasmic Es*β*-catenin; Ⅱ: nuclear Es*β*-catenin) by Western blot; (Ⅲ) The corresponding gray value analysis of Western blot. *E*, immunofluorescence analysis detected the expression levels of Es*β*-catenin protein in hemocytes of crabs at 0 h and 96 h after EsGSK3β knockdown. *F*, the effect of EsGSK3β knockdown on Es*β*-catenin target gene EsAXIN-1, EsAP-1, EsMMP2, and EsMMP14 transcription. DAPI, 4′-6-diamidino-2-phenylindole dihydrochloride; PCR; dsRNA, double-stranded RNA; qRT–PCR, quantitative real-time reverse transcription.

To further analyze the relationship between EsGSK3β and Es*β*-catenin. Western blotting, immunocytochemical assays, and qRT–PCR were used to analyze the effect of EsGSK3*β* knockdown by sequence-specific RNAi on Es*β*-catenin and target gene transcription. First, the transcription of EsGSK3β was detected after RNAi by qRT–PCR assays, and the results showed that EsGSK3β interference in the EsGSK3β-dsRNA treatment group was effective when compared to the control group and lasted for 96 h (Fig. 3*A*). Western blot and immunocytochemical assays also showed the same results, and EsGSK3β activity from EsGSK3β-Tyr214 was also significantly downregulated (Fig. 3, *B* and *C*). These results indicated that EsGSK3β knockdown had a negative effect on EsGSK3β activity. Second, to further explore the effect of repressed EsGSK3β expression on Es*β*-catenin, Western blot and immunocytochemical assays were carried out. The results showed that after knockdown of EsGSK3β, the quantity of Es*β*-catenin was significantly increased both in the cytoplasm and nucleus (Fig. 3, *D* and *E*). Consistent with this result, the transcription levels of the Es*β*-catenin target genes Axin-1, AP-1, MMP2, and MMP14 were all significantly upregulated compared with those in the control group when EsGSK3β was knocked down (Fig. 3*F*). Altogether, our results strongly suggest that in crabs, enhancing the expression of EsGSK3β can significantly accelerate the degradation of Es*β*-catenin. During the process of *S. eriocheiris* infecting the crab, EsGSK3β activity was significantly upregulated, and accelerated Es*β*-catenin degradation restrained the host cell Wnt-*β*-catenin signaling pathway.

### The Wnt-β-Catenin Signaling Pathway Positively Regulates the Innate Immune Response in Crab Hemocytes In Vivo

**Fig. 4:**
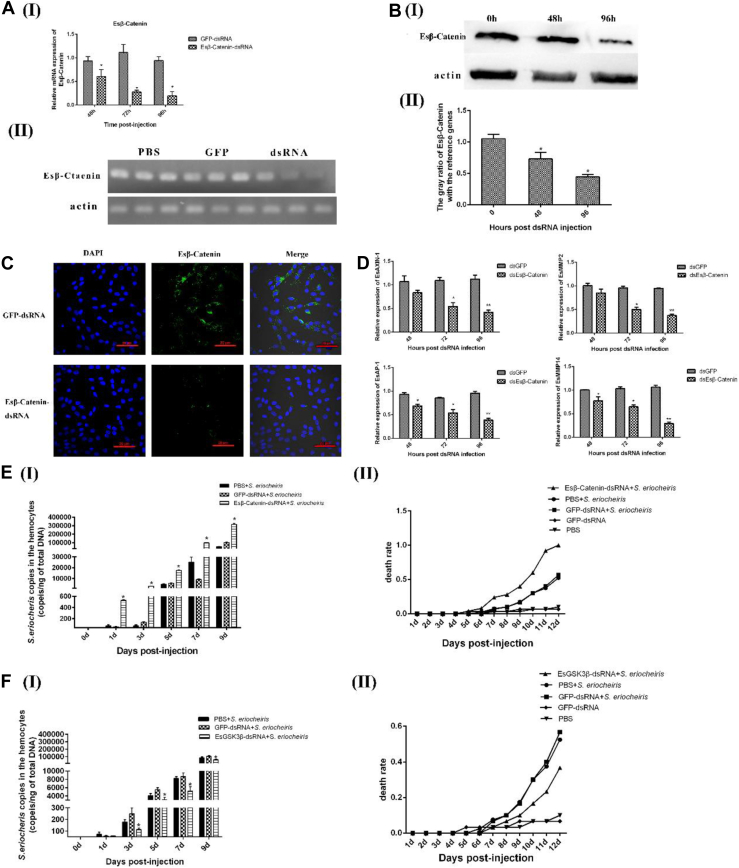
**The Wnt-*β*-catenin pathway positively regulates the crab innate immune response *in vivo*.***A*, *B*, *C*, qRT–PCR (*A*), Western blot (*B*), and immunofluorescence (*C*) analyses after Es*β*-catenin knockdown. *D*, qRT–PCR analysis of the effect of Es*β*-catenin knockdown on the transcription of the target genes EsAXIN-1, EsAP-1, EsMMP2, and EsMMP14. *E*, the copies of *S. eriocheiris* in crab hemocytes throughout the infection course after Es*β*-catenin RNAi (I). The death rate statistics of Es*β*-catenin-silenced crabs after *S. eriocheiris* infection. GFP-dsRNA + *S. eriocheiris*, PBS + *S. eriocheiris*, PBS, and GFP-dsRNA as control groups (II). *F*, The copies of *S. eriocheiris* in crab hemocytes throughout the infection course after EsGSK3β RNAi (I). The death rate statistics of EsGSK3β-silenced crabs after *S. eriocheiris* infection. GFP-dsRNA + *S. eriocheiris*, PBS + *S. eriocheiris*, PBS, and GFP-dsRNA as control groups (II). Statistical significance is indicated (∗, *p* < 0.05). The data are the means ± SD for three independent experiments. DAPI, 4′-6-diamidino-2-phenylindole dihydrochloride; PCR, dsRNA, double-stranded RNA; qRT–PCR, quantitative real-time reverse transcription.

To further confirm the function of the crab Wnt pathway in *S. eriocheiris* infection *in vivo*, Es*β*-catenin and EsGSK3β were knocked down by sequence-specific RNAi. qRT–PCR, Western blot, and immunofluorescence assay results showed that the expression level of Es*β*-catenin was significantly repressed in Es*β*-catenin-dsRNA–treated crabs, but there were no obvious changes in the control groups (Fig. 4, *A*–*C*). The EsGSK3β RNA interference efficiency was detected as described above. To further verify the effect of low Es*β*-catenin transcription on the downstream target genes, Axin-1, AP-1, MMP2, and MMP14 were selected and verified by qRT–PCR. In contrast to the results of EsGSK3β knockdown, repression of Es*β*-catenin by RNAi significantly downregulated the transcription levels of those target genes (Fig. 4*D*). As shown in Figure 4*E* Ⅰ and Ⅱ, the copies of *S. eriocheiris* in the Es*β*-catenin-dsRNA + *S. eriocheiris* group was obviously higher than the control groups from 1 day to 9 days. The cumulative mortality of the Es*β*-catenin-dsRNA + *S. eriocheiris* group was also significantly higher than that of the control groups during the period of 6 days to 12 days. As we expected, the copies of *S. eriocheiris* and cumulative mortality of crabs in the EsGSK3*β*-dsRNA + *S. eriocheiris* group were significantly lower than that in the control group (Fig. 4*F*Ⅰ and Ⅱ). Altogether, the results strongly suggest that the stabilization and/or accumulation of Es*β*-catenin is necessary to enhance the antibacterial immunity of crabs.

### A GSK3β Inhibitor (SB-216763) Enhances the Innate Response of Crabs by Increasing Esβ-Catenin Stabilization *In Vitro*

**Fig. 5:**
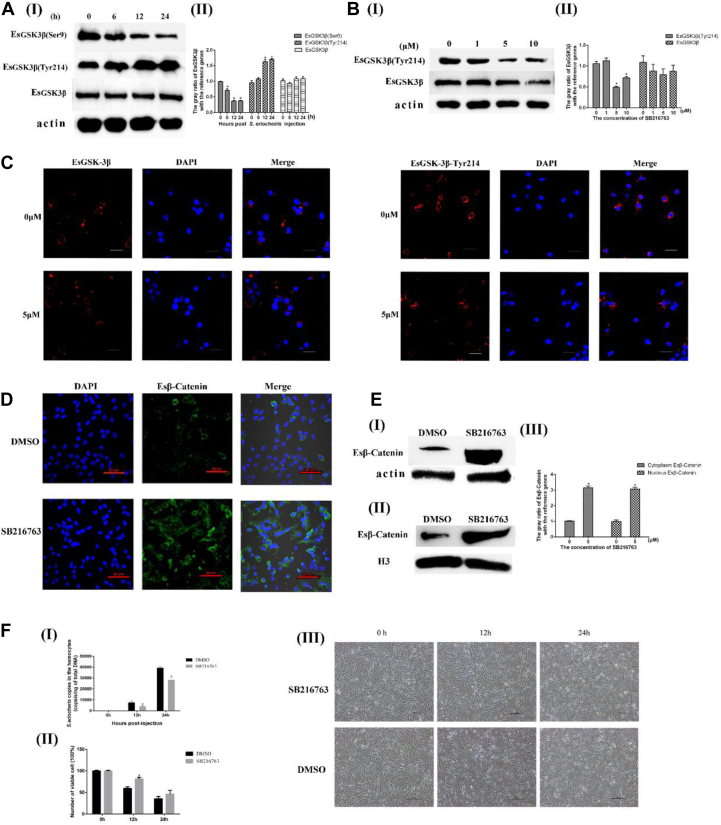
**The role of the Wnt-*β*-catenin pathway during *S. eriocheiris* infections in hemocytes *in vitro*.***A*, Western blot analysis of the expression and activity of EsGSK3β in hemocytes after *S. eriocheiris* infection *in vitro* (I). The corresponding *gray* value analysis of Western blot (II). *B*, Western blot analysis of the expression of EsGSK3β and EsGSK3β-Tyr214 in hemocytes treated with different concentrations (0, 1, 5, and 10 μM) of SB216763; DMSO-treated cells were used as the negative control; Actin was used as the reference protein (I). The corresponding *gray* value analysis of Western blot (Ⅱ). *C*, immunofluorescence analysis of the expression of EsGSK3β and EsGSK3β-Tyr214 in hemocytes treated with SB216763. DMSO-treated cells were used as the negative control. Bar: 10 μM. *D*, immunofluorescence analysis of Es*β*-catenin expression in hemocytes treated with SB216763; DMSO-treated cells were used as the negative control. *E*, Western blot analysis of Es*β*-catenin expression in the hemocyte cytoplasm (I) and nucleus (II) after SB216763 treatment. *F*, the copies of *S. eriocheiris* in the hemocytes (I), the cell viability determined by CCK-8 (II), and the morphology of hemocytes after *S. eriocheiris* infection (III). SB216763 (5 μM) was used as the experimental group, and DMSO was used as the negative control group. Statistical significance is indicated (∗, *p* < 0.05). The data are the means ± SD for three independent experiments. CCK-8, cell counting kit-8; DAPI, 4′-6-diamidino-2-phenylindole dihydrochloride.

To further elucidate the function of the Wnt pathway, EsGSK3β expression in hemocytes during *S. eriocheiris* infection was detected using Western blotting. When *S. eriocheiris* infected the hemocytes of crabs, the expression level of EsGSK3β was not significantly changed, but the activity of EsGSK3β was upregulated significantly (*i.e.,* EsGSK3β-Ser9 was downregulated, and EsGSK3β-Tyr214 was upregulated) (Fig. 5*A*). These results were similar to the results obtained during *S. eriocheiris* infection of crab hemocytes *in vivo*. SB-216763 is an ATP-competitive inhibitor of GSK3β that can specifically downregulate the activity of GSK3β (previous studies also showed that this inhibitor can downregulate the phosphorylation level of GSK3β Tyr214) and then activate the Wnt-*β*-catenin signaling pathway, ultimately increasing Es*β*-catenin stabilization. Western blot and immunocytochemical assay results showed that 5 μM SB-216763 was the optimum concentration to activate the crab hemocyte Wnt-*β*-catenin signaling pathway (Fig. 5*B*). Similarly, the inhibition of EsGSK3β catalytic activity by SB-216763 resulted in Es*β*-catenin cytoplasmic accumulation and nuclear translocation (Fig. 5, *C* and *D*). We then investigated the role of EsGSK3β in the negative regulation of host cell innate immunity. As expected, compared with the DMSO group, the number of copies of *S. eriocheiris* in the SB-216763 (5 μM)-treated group was significantly decreased (Fig. 5*E*Ⅰ). According to the CCK-8 test, the viability of hemocytes in the SB-216763 (5 μM) group was obviously higher than that in the DMSO-treated group (Fig. 5*E*Ⅱ). The cellular morphology corresponded to the results of the CCK-8 test (Fig. 5*E*III). Altogether, the results strongly suggest that EsGSK3β plays a key role in catalytic activity in negatively regulating the crab antimicrobial innate immune response mediated by the Wnt-*β*-catenin signaling pathway.

### EsGSK3β Through Regulated Esβ-Catenin Phosphorylation Affect the S2 Cell-Resist S. eriocheiris Infection

**Fig. 6:**
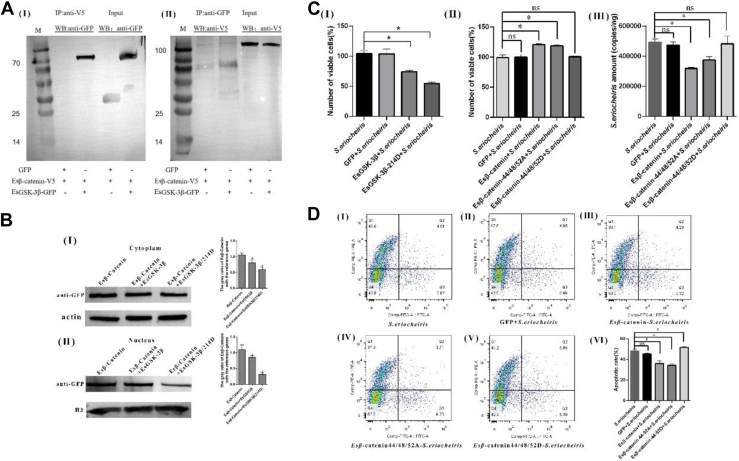
**EsGSK3β through inhibiting Es*β*-catenin nuclear transfer effect the S2 cell against *S. eriocheiris* infection.***A*, Co-IP results showed the interaction between EsGSK3β and Es*β*-catenin in S2 cells. Cell lysates were immunoprecipitated with anti-GFP and anti-V5 antibodies, followed by Western blot analysis. *B*, Western blot analysis of Es*β*-catenin expression in the S2 cell cytoplasm (I) and nucleus (II) after pAc5.1-Es*β*-catenin-V5 cotransfection with pAc5.1-GFP, pAc5.1-EsGSK3β, and pAc5.1-EsGSK3β/214D 48 h. *C*, after *S. eriocheiris* infection 48 h, CCK-8 detected the cell viability of S2 cell which transfect with the diffident recombinant plasmid (I) pAc5.1-GFP, pAc5.1-EsGSK3β and Ac5.1-EsGSK3β/214D and (II) pAc5.1-GFP, pAc5.1-Es*β*-catenin, pAc5.1-Es*β*-catenin/44/48/52A, and pAc5.1-Es*β*-catenin/44/48/52D; the S2 cell *S. eriocheiris* copies after transfection with the diffident recombinant plasmid (III). *D*, the flow cytometry analysis the S2 cell apoptotic rate which transfect with the diffident recombinant plasmid, (I) *S. eriocheiris*, (II) GFP + *S. eriocheiris*, (III) Es*β*-catenin + *S. eriocheiris*, (Ⅳ) Es*β*-catenin/44/48/52A + *S. eriocheiris*, (Ⅴ) Es*β*-catenin/44/48/52D + *S. eriocheiris,* and (Ⅵ) the apoptosis rate of each group statistically analysis.

To further analyze whether the EsGSK3β through regulated Esβ-catenin phosphorylation affect the host cell-resist *S. eriocheiris* infection, the recombinant plasmids pAc5.1-EsGSK3β-GFP, pAc5.1-EsGSK3β-V5, pAc5.1-Esβ-catenin-GFP, and pAc5.1-Esβ-catenin-V5 were successfully constructed (supplemental Fig. S3). Then, a coimmunoprecipitation assay was performed in *Drosophila* S2 cells (Fig. 6*A*). The results showed that the EsGSK3β could interact with Esβ-catenin, and the control tag protein (GFP and V5) could not interact with EsGSK3β or Esβ-catenin. Further analysis showed co-expression EsGSK3β and Esβ-catenin in S2 cell, and the EsGSK3β could significantly reduce the Esβ-catenin expression level both in cytoplasm and nucleus compared with the control group (Fig. 6*B*). And co-expression EsGSK3β/214D and Esβ-catenin in S2 cell, the cytoplasm and nuclear Esβ-catenin level was further reduced compared with the other two group (only transfected pAc5.1-Esβ-catenin-GFP cells and co-transtected pAc5.1-Esβ-catenin-GFP/pAc5.1-EsGSK3β/214D cells). CCK-8 analysis showed that the ability of S2 cells to resist *S. eriocheiris* infection was significantly reduced after overexpression EsGSK3β (Fig. 6*C*Ⅰ). In the Esβ-catenin and Esβ-catenin/44/48/52A overexpression group, the cell viability significantly enhanced compared with the control group, while in the Esβ-catenin/44/48/52A overexpression group, there was none significant change on cell viability. Consistent with those results, theS2 cell *S. eriocheiris* copies were significantly reduced after overexpression Esβ-catenin and Esβ-catenin/44/48/52A, and no significant change was observed in Esβ-catenin/44/48/52A overexpression group (Fig. 6*C*Ⅱ). The flow cytometry analysis showed that the cell apoptotic rate of overexpression Esβ-catenin and Esβ-catenin/44/48/52A group was significantly lower than control group, and no significant change in Esβ-catenin/44/48/52A overexpression group was observed (Fig. 6*D*). In conclusion, the results show that EsGSK3β could directly interact with Esβ-catenin regulating the Esβ-catenin stability and nuclear transfer and then effected the host against *S. eriocheiris* infection, and in this process the two key proteins amino phosphorylation played an important role.

### Crab Wnt-β-Catenin Signaling Pathway Crosstalk With the Toll Signaling Pathway Regulates Hemocyte AMP Transcription

**Fig. 7:**
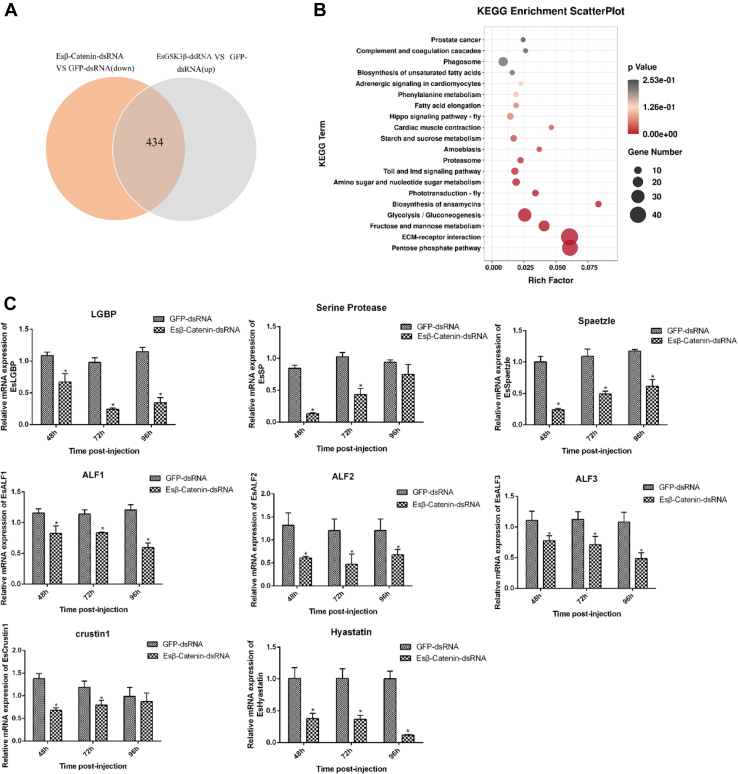
**Transcriptomic analysis of Wnt-*β*-catenin pathway-related innate immune genes.***A*, Venn diagram of the overlap of differentially expressed genes from Es*β*-catenin-dsRNA *versus* GFP-dsRNA (significantly downregulated) and EsGSK3β-dsRNA *versus* GFP-dsRNA (significantly upregulated) screening. *B*, KEGG analysis of overlapping differentially expressed genes. *C*, expression profiles of selected genes from Venn analysis by qRT–PCR. Statistical significance is indicated (∗*p* < 0.05). The data are the means ± SD for three independent experiments. KEGG, Kyoto Encyclopedia of Genes and Genomes; dsRNA, double-stranded RNA.

Target innate immune–related genes whose transcription was induced by Es*β*-catenin and whose transcription was suppressed by EsGSK3β were identified. Venn analysis of differentially transcribed genes from Es*β*-catenin-dsRNA *versus* GFP-dsRNA (significantly downregulated) and EsGSK3β-dsRNA *versus* GFP-dsRNA (significantly upregulated) of the screening generated 434 candidates (Fig. 7*A*). KEGG enrichment analysis of those candidate genes showed that many innate immune–related genes belonged to the Toll and IMD signaling pathways, including LGBP, SP, spaetzle protein, and several important AMPs (Fig. 7*B*, supplemental Table S8). To further validate these differentially transcribed immune-related genes, qRT–PCR analyses were carried out. The results showed that the transcription levels of LGBP, SP, spaetzle, ALF1, ALF2, ALF3, crustin 1, and hyastatin were all significantly downregulated after knockdown of Es*β*-catenin expression by RNAi (Fig. 7*C*).

## Discussion

*S. eriocheiris*, one of the smallest prokaryotes and intracellular pathogens, has been identified as the pathogen of TD, a devastating neurological epizootic of crustaceans (6, 7). Previous studies indicates that the hemocytes (the principal immune effector cells) and thoracic ganglion (the neural control center for appendage movement) of crustaceans are the two main target tissues. *S. eriocheiris* infection of the thoracic ganglion is the direct reason for typical paroxysmal tremors of crab pereiopods. However, the molecular pathogenesis underlying this particular crustacean neuropathic disease remains poorly understood. To identify the pathway or process associated with TD caused by *S. eriocheiris* infection, 680 significantly differentially expressed phosphorylated proteins were obtained in TD-affected crabs thoracic ganglions *via* the phosphorylproteome. The Wnt pathway was restrained, corresponding to many nervous system developments, and signal transmission-related pathways were also disrupted.

Extensive research has established that dysregulation of the Wnt pathway causes a variety of neurological diseases, such as AD, Pick’s disease, and corticobasal degeneration (3, 4). The Wnt-*β*-catenin pathway, as the canonical Wnt pathway, is best characterized. In the absence of Wnt ligands, *β*-catenin is phosphorylated by GSK3β, marking it for ubiquitin-mediated proteasomal degradation (21, 22, 23, 24). GSK3β is a highly conserved serine/threonine protein kinase. The activity of GSK3β depends on the phosphorylation levels at the inactivating form Ser9 site and the activated form Tyr214 (Tyr216 in humans). Upon Wnt pathway activation, the dishevelled (DVL) proteins transduce signals that inhibit GSK3β activity (*via* Ser9 phosphorylation) and stabilize cytoplasmic *β*-catenin, enabling its nucleus translocation to mediate the expression of many genes, such as *α7-nicotinic AchR, Bcl-2*, and *neuroligin* (25, 26, 27, 28). In the case of AD, the Wnt-*β*-catenin pathway has been shown to be restrained in the brain cells of AD patients, thus causing the apoptosis and degeneration of neuronal cells (29). Similar to the results of AD, the phosphorylproteome results revealed that when *S. eriocheiris* infected the thoracic ganglion of crabs, the phosphorylation level of EsDVL was significantly downregulated, and the corresponding phosphorylation level of EsGSK3β-Ser9 was also significantly downregulated. These results strongly suggest that *S. eriocheiris* infection can enhance the activity of EsGSK3β, thereby suppressing the Wnt-*β*-catenin pathway in the crab neural tissue. A major biochemical and structural characteristic of neurodegenerative tauopathies is abnormal tau hyperphosphorylation, which by leading to neuronal degeneration caused multiple neurological disorders (3, 4, 30, 31, 32). Tau protein contains phosphorylation sites targeted by key kinases, including p38 kinases, MARK, and GSK3β (33, 34, 35). Similar to most neurological diseases, abnormal tau hyperphosphorylation at two phosphorylation sites (Ser77 and Ser284) was observed in the diseased crab nerve tissue. Notably, GSK3β can phosphorylate tau at most serine and threonine residues (more than 30 distinct phosphorylation sites to date) (36, 37). In AD brains, PP2A/B is the most active phosphatase in dephosphorylating hyperphosphorylated tau (38, 39). In the current study, the phosphorylation of PP2B was significantly downregulated upon *S. eriocheiris* infection. In summary, *S. eriocheiris* infection can enhance the activity of protein kinases (EsGSK3β, EsMAPK, and Esp38) and downregulate phosphatase (PP2B), and this imbalance drives pathological tau hyperphosphorylation at Ser77 and Ser284, ultimately leading to neural cytoskeletal destabilization thereby destroying the nervous systems of crabs. With the Wnt ligands, the Wnt-Ca^2+^ signaling pathway can increase the intracellular level of Ca^2+^ through DVL and induce the activation of calcium-sensitive kinases, CaMKII and protein kinase C (PKC) (40). PKC inhibits GSK3*β* through Ser9 phosphorylation and induces two main consequences: stabilizing cytoplasmic *β*-catenin and reduced pathological phosphorylation of tau protein (41, 42). Inositol 1,4,5-triphosphate receptor, also known as IP3R or InsP3R, an endoplasmic reticulum–localized calcium release channel, serves as a critical regulator of intracellular Ca^2+^ signaling. Phosphorylation of IP3R by PKA regulates the sensitivity of IP3R to IP3, thereby regulates Ca^2+^ release dynamics (43, 44, 45, 46). In the present study, phosphoproteomic analysis revealed that the phosphorylation of PKC, PKA, IP3R, and CaMKII was significantly downregulated upon *S. eriocheiris* infection. The downregulation of PKC phosphorylation leads to an increase in EsGSK3β activity, increased degradation of cytoplasmic Es*β*-catenin, and abnormal tau hyperphosphorylation, which ultimately disrupts the crab nerve system.

Neurotransmitters released from synaptic vesicles are mediated by complex machinery. Soluble N-ethylmaleimide-sensitive factor attachment protein receptors (SNAREs) play important roles in neurotransmitter release. v-SNAREs are located on the vesicle membrane and contain VAMP and SYT, while t-SNAREs on the target membrane are composed of presynaptic plasma membrane proteins SYN and SNAP-25. The assembled v-SNARE/t-SNARE complex contains four helices, three of which are supplied by t-SNARE and one by v-SNARE, which play a decisive role in synaptic vesicle membrane docking and fusion (47, 48). The regulation of synaptic activity can be controlled by protein phosphorylation levels through phosphatases and kinases. Casein kinase II, PKA, PKC, and CaMKII are all involved in different aspects of synaptic efficacy in long-term changes (49, 50). For example, SYN and SNAP-25 were phosphorylated by casein kinase II and PKA, respectively. In the current study, quantitative phosphoproteomics revealed that during the symptomatic phase of *S. eriocheiris* infection (characterized by paroxysmal pereiopod tremors), the phosphorylation levels of VAMP, SNAP-25, SYT, and SYN were significantly downregulated. Meanwhile, the phosphorylation levels of several protein kinases also significantly downregulated. The above results suggest that the abnormal phosphorylation of VAMP, SNAP-25, SYT, and SYN has a negative impact on the interaction between v-SNARE/t-SNARE to affect neurotransmitter release from synaptic vesicles. By LC–MS/MS analysis, four kinds of important neurotransmitters (5-hydroxy-L-tryptophan, serotonin, acetylcholine, and γ-amino-butyric acid) were significantly changed in *S. eriocheiris*-infected crabs exhibiting tremor symptoms. To date, many studies have suggested that dysregulated neurotransmitter metabolism is related to many kinds of neurological diseases (51, 52, 53, 54). From the above, *S. eriocheiris* could induce several key proteins (VAMP, SNAP-25, SYT, and SYN, etc.) abnormal phosphorylation, which had a negative impact on neurotransmitter metabolism thereby inducing neurotransmitter metabolic disorders, and causing paroxysmal tremors of crab pereiopods.

The hemocytes of crustaceans, as the most important immune cell and also the first target cell of *S. eriocheiris* infection, play critical roles in the host against this pathogen infection. In the current study, the role of the Wnt-*β*-catenin pathway in *S. eriocheiris* infecting crab hemocytes was investigated *in vivo* and *in vitro*. The results showed that during *S. eriocheiris* infection of the hemocytes of crabs *in vivo*, the core protein of the Wnt-*β*-catenin pathway Es*β*-catenin’s cytoplasmic accumulation and nuclear translocation were restrained. Meanwhile, the expression level of EsGSK3β (activated form EsGSK3β-Tyr214 increased and inactivated form EsGSK3β-Ser9 decreased) was upregulated in response to *S. eriocheiris* infection. Research on model organisms have clearly demonstrated that GSK3β serves as a negative regulator of the Wnt-*β*-catenin pathway and that it enhances the activity of GSK3β to accelerate *β*-catenin degradation (21, 22). However, in crustaceans, the relationship between GSK3β and *β*-catenin is still unclear. To further analyze the effect of EsGSK3β on Es*β*-catenin stabilization in crabs, knockdown of the EsGSK3*β* gene by RNAi (total EsGSK3*β* and EsGSK3*β*-Tyr214 expression levels were all downregulated) resulted in Es*β*-catenin cytoplasmic accumulation and nuclear translocation, and Es*β*-catenin target gene transcription was upregulated. These results demonstrated that EsGSK3β could negatively regulate the stabilization of Es*β*-catenin, similar to model organisms. Altogether, these results strongly suggested that *S. eriocheiris* subverted host defense by upregulation of EsGSK3β catalytic activity, subsequent acceleration of Es*β*-catenin degradation, and suppression of the crab Wnt-*β*-catenin signaling pathway. Similar results were also obtained in *S. eriocheiris*-infected crab hemocytes *in vitro* and the thoracic ganglion of crabs. Similar to our results, when *S. typhimurium* infected colon epithelial cells, GSK3β-dependent *β*-catenin degradation was upregulated, which suppressed the Wnt-*β*-catenin pathway to facilitate the pathogen infection (55, 56). However, the effect of *S. eriocheiris* restraining the crab Wnt-*β*-catenin signaling pathway on its infection is unclear. When Es*β*-catenin expression was knocked down in crab hemocytes, the copy number of *S. eriocheiris* and the corresponding cumulative mortalities of Es*β*-catenin-dsRNA-treated crabs were increased. In contrast, knockdown of EsGSK3β by RNAi resulted in a significantly downregulated copy number of *S. eriocheiris* and cumulative mortality of crabs. These results suggest that the crab Wnt-*β*-catenin pathway played a positive role in hemocytes resisting *S. eriocheiris* infection. Similarly, when the Wnt-*β*-catenin pathway was restrained in *Mus japonicus* by knockdown of *β*-catenin, the clearance abilities of the host against gram-positive, gram-negative bacteria and viruses were impaired (57). *In vitro*, when hemocytes were treated with *S. eriocheiris*, the Wnt-*β*-catenin pathway was also restrained, similar to the results *in vivo*. Pretreatment of hemocytes with the GSK3β inhibitor SB-216763 (mimicking activation of the Wnt-*β*-catenin signaling pathway) *in vitro* successfully inhibited the activity of EsGSK3β and then increased Es*β*-catenin cytoplasmic accumulation and nuclear translocation, resulting in significantly improved host cell antibacterial ability. Meanwhile, compared with the control group, the hemocytes viability and the copy number of *S. eriocheiris* in SB-216763-pretreated group significantly decreased. Similar results also found that host resistance against *Pseudomonas aeruginosa* infection was promoted in the treatment of mouse cell with an inhibitor of GSK3β, a compound that mimics Wnt-*β*-catenin pathway activation by inhibition of GSK3β activity (58). Our results in the present study revealed that the EsGSK3β by direct interaction with Esβ-catenin regulated the Esβ-catenin stability and nuclear transfer and then effected the host against *S. eriocheiris* infection, in this process the two proteins key amino phosphorylation played an important role. Taken together, the above results demonstrated that the Wnt-*β*-catenin signaling pathway played a crucial role in the crustacean innate immune defense against *S. eriocheiris* infection. When infecting crab hemocytes, *S. eriocheiris* could upregulate EsGSK3β activity, accelerate Es*β*-catenin degradation, restrain the host cell Wnt-*β*-catenin signaling pathway, and then downregulate the host cell innate immune antibacterial response to help with infection.

To date, many studies have found that the Wnt pathway participates in regulating innate immunity by direct or crosstalk with other pathways. A genome-wide RNAi screen revealed that the Wnt-*β*-catenin signaling pathway is involved in regulating the expression of IFNB1, IFIT1, and TNF in a *β*-catenin-dependent effector mechanism in HEK 293T cells upon Sendai virus infection (59). Wnt-*β*-catenin pathway crosstalk with the Toll pathway regulated the mouse macrophage inflammatory response in a Wnt3a-dependent manner (60). Toll-like receptor 2 crosstalk with the Wnt-*β*-catenin signaling pathway regulates cytokine production in megakaryocytes (61). *Drosophila* WntD is upregulated when flies face *Listeria monocytogenes* infection *via* the Toll signaling pathway and is involved in host AMP expression regulation (62). In *L. vannamei*, *β*-catenin induced activation of the AMP promoter to help the host defend against invading pathogens, but the mechanism is unclear (15). In the present study, transcriptomic analysis revealed that several Toll pathway genes and AMP transcription were induced by Es*β*-catenin and suppressed by EsGSK3β, including LGBP, several kinds of serine proteinases, spaetzle, ALF1/2/3, crustin1, and hyastatin. The Toll pathway plays an essential role in regulating cell AMP transcription. When pattern recognition receptors recognize the corresponding foreign signal, the spaetzle protein is activated with a serine proteinase cascade reaction. The activated spaetzle binds to Toll receptor, activating the Toll signaling pathway and promoting the transcription of AMP genes (63). Altogether, our results strongly demonstrated that the Wnt-*β*-catenin signaling pathway could activate spaetzle protein in an LGBP/serine proteinase/spaetzle manner and crosstalk with the Toll signaling pathway to regulate crab AMP transcription. When *S. eriocheiris* infects the hemocytes of crabs, this bacterium employs increase the phosphorylation activity of EsGSK3β to restrain the Wnt-*β*-catenin signaling pathway, reduce the transcription and release of AMPs in hemocytes, and then help itself infect and escape the host immune response both *in vivo* and *in vitro*.

**Fig. 8:**
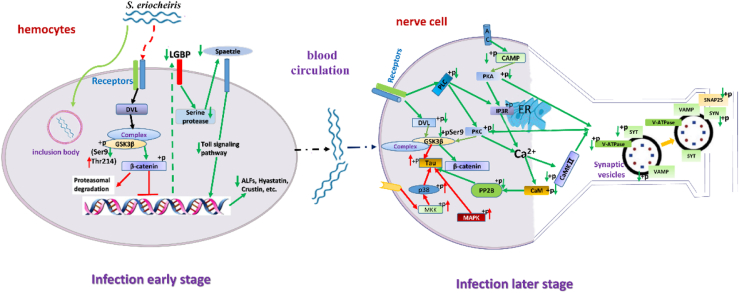
**A schematic diagram was drawn to describe the *S. eriocheiris*-infected hemocytes and the thoracic ganglion of crabs.** For abbreviations and explanation, see the text.

In summary, our study establishes that the Wnt pathway plays a critical role in *S. eriocheiris* infection and causes neuropathic diseases in crustaceans. At the early stage of infection, when *S. eriocheiris* infects crab hemocytes, this intracellular bacterium can enhance the activity of EsGSK3β, accelerate Es*β*-catenin degradation, and restrain the host cell Wnt-*β*-catenin signaling pathway. The restrained Wnt-*β*-catenin pathway could crosstalk with the Toll signaling pathway in an LGBP/SP/spaetzle manner and then downregulate crab AMP transcription. At the later stage, with blood circulation, *S. eriocheiris* can infect crab nerve tissue, enhance EsGSK3β phosphorylation activity to restrain the Wnt pathway in crab nerve tissue, induce abnormal tau hyperphosphorylation, cause abnormal neurotransmitter release–associated protein phosphorylation levels, and finally disturb the crab nervous system and transmit signals while causing pereiopod tremors (Fig. 8). This is the first report about the molecular mechanisms of neurogenic disease in crustaceans caused by intracellular bacteria. Our results in this article provide novel insights to help us understand the relationship between the Wnt pathway in crustaceans and neurological disease caused by intracellular bacteria.

## Data availability

All data needed to evaluate the conclusions in the paper are present in the paper and the supplementary materials. The mass spectrometry proteomics data have been deposited to the ProteomeXchange Consortium (http://proteomecentral.proteomexchange.org) *via* the PRIDE partner repository with the dataset identifier PXD022892 (the Reviewer account details: Username: reviewer_pxd022892@ebi.ac.uk; Password: UcO7c6aI). Annotated spectra to support the identification of phosphorylated peptides from the TMT phosphoproteome are available in the source data section of this submission. The transcriptomic sequencing date have been deposited to the GEO of NCBI with the GEO accession numbers GSE207959 (https://www.ncbi.nlm.nih.gov/geo/query/acc.cgi?acc=GSE207959).

## Supplemental data

This article contains supplemental data.

## Conflict of Interest

The authors declare no competing interests.

## References

[1] Ciani L., Salinas P.C. (2005). WNTs in the vertebrate nervous system: from patterning to neuronal connectivity. Nat. Rev. Neurosci..

[2] Inestrosa N.C., Arenas E. (2011). Emerging roles of Wnts in the adult nervous system. Nat. Rev. Neurosci..

[3] Grundke-Iqbal, Iqbal I., Tung K.Y.C., Quinlan M., Wisniewski H.M., Binder L.I. (1986). Abnormal phosphorylation of the microtubule-associated protein tau (tau) in Alzheimer cytoskeletal pathology. Proc. Natl. Acad. Sci. U. S. A..

[4] Lee V.M., Balin B.J., Otvos L.J., Trojanowski J.Q. (1991). A68: a major subunit of paired helical filaments and derivatized forms of normal Tau. Science.

[5] Zhang Y., Bao H., Miao F., Peng Y., Shen Y., Gu W. (2015). Production and application of polyclonal and monoclonal antibodies against *Spiroplasma eriocheiris*. Sci. Rep..

[6] Wang W., Gu Z. (2002). Rickettsia-like organism associated with tremor disease and mortality of the Chinese mitten crab *Eriocheir sinensis*. Dis. Aquat. Org..

[7] Wang W., Gu W., Gasparich G.E., Bi K., Ou J., Meng Q. (2011). *Spiroplasma eriocheiris* sp. Nov., associated with mortality in the Chinese mitten crab, *Eriocheir sinensis*. Int. J. Syst. Evol. Microbiol..

[8] Liang T.M., Li X.L., Du J., Yao W., Sun G.Y., Dong X.H. (2011). Wen Identification and isolation of a spiroplasma pathogen from diseased freshwater prawns, *Macrobrachium rosenbergii*, in China: a new freshwater crustacean host. Aquaculture.

[9] Bi K., Huang H., Gu W., Wang J., Wang W. (2008). Phylogenetic analysis of Spiroplasmas from three freshwater crustaceans (*Eriocheir sinensis, Procambarus clarkia* and *Penaeus vannamei*) in China. J. Invertebr Pathol..

[10] Zeigel R.F., Clark H.F. (1974). Electron microscopy of the suckling mouse cataract agent: a noncultivable animal pathogen possibly related to Mycoplasma. Infect. Immun..

[11] Wang W., Rong L., Gu W., Du K., Chen J. (2003). Study on experimental infections of *Spiroplasma* from the Chinese mitten crab in crayfish, mice and embryonated chickens. Res. Microbiol..

[12] Hou L., Liu Y., Gao Q., Xu X., Ning M., Bi J. (2017). *Spiroplasma eriocheiris* adhesin-like protein (ALP) interacts with EGF Domain proteins to facilitate infection. Front. Cell. Infect. Microbiol..

[13] Wang W., Zhu N., Gu Z., Du K., Xu Z. (2002). Study on the transmission of tremor disease (TD) in the Chinese mitten crab, *Eriocheir sinensis* (Crustacea: decapoda). J. Invertebr. Pathol..

[14] Silva-Garcia O., Valdez-Alarcon J.J., Baizabal-Aguirre V.M. (2014). The Wnt/beta-catenin signaling pathway controls the inflammatory response in infections caused by pathogenic bacteria. Mediat. Inflamm..

[15] Zhang S., Shi L., Li H., Li S., Wang J., Li C. (2016). Cloning, identification and functional analysis of a *β*-catenin homologue from Pacific white shrimp, *Litopenaeus vannamei*. Fish Shellfish Immunol..

[16] Ding Z., Tang J., Xue H., Li J., Gu W., Meng Q. (2014). Quantitative detection and proliferation dynamics of a novel *Spiroplasma eriocheiris* pathogen in the freshwater crayfish, *Procambarus clarkii*. J. Invertebr. Pathol..

[17] Hou L., Ma Y., Cao X., Gu W., Cheng Y., Wu X. (2020). Transcriptome profiling of the *Eriocheir sinensis* thoracic ganglion under the *Spiroplasma eriocheiris* challenge. Aquaculture.

[18] Liu P., Zheng H., Meng Q., Terahara N., Gu W., Wang S. (2017). Chemotaxis without conventional two-component System, based on cell polarity and aerobic conditions in helicity-switching swimming of *Spiroplasma eriocheiris*. Front. Microbiol..

[19] Park C.H., Lee G.H., Ahn S.G., Yoon J.H., Oh S.H. (2013). Serine 9 and Tyrosine 216 phosphorylation of GSK-3β differentially regulates autophagy in acquired cadmium resistance. Toxicol. Sci..

[20] Graham J.R., Tullai J.W., Cooper G.M. (2010). GSK-3 represses growth factor-inducible genes by inhibiting NF-kappaB in quiescent cells. J. Biol. Chem..

[21] Kohn A.D., Moon R.T. (2005). Wnt and calcium signaling: β-catenin-independent pathways. Cell Calcium..

[22] Gao C., Chen Y.G. (2010). Dishevelled: the hub of Wnt signaling. Cell. Signal..

[23] van Amerongen R., Nusse R. (2009). Towards an integrated view of Wnt signaling in development. Development.

[24] Wallingford J.B., Habas R. (2005). The developmental biology of dishevelled: an enigmatic protein governing cell fate and cell polarity. Development.

[25] Fuenzalida K., Quintanilla R., Ramos P.D., Piderit, Fuentealba R.A., Martinez G. (2007). Peroxisome proliferatoractivated receptor gamma up-regulates the Bcl-2 anti-apoptotic protein in neurons and induces mitochondrial stabilization and protection against oxidative stress and apoptosis. J. Biol. Chem..

[26] Farías G.G., Vallés A.S., Colombres M., Godoy J.A., Toledo E.M., Lukas R.J. (2007). Wnt-7a induces presynaptic colocalization of α7-nicotinic acetylcholine receptors and adenomatous polyposis coli in hippocampal neurons. J. Neurosci..

[27] Arrázola M.S., Varela-Nallar L., Colombres M., Toledo E.M., Cruzat F., Pavez L. (2009). Calcium/calmodulin-dependent protein kinase type IV (CaMKIV) is a target gene of the Wnt/β-catenin signaling pathway. J. Cell Physiol..

[28] Hödar C., Assar R., Colombres M., Aravena A., Pavez L., González M. (2010). Genome-wide identification of new Wnt/beta-catenin target genes in the human genome using CART method. BMC Genomics.

[29] Inestrosa N.C., Toledo E.M. (2008). The role of Wnt signaling in neuronal dysfunction in Alzheimer’s disease. Mol. Neurodegener..

[30] Wang J.Z., Grundke-Iqbal I., Iqbal K. (2007). Kinases and phosphatases and tau sites involved in Alzheimer neurofibrillary degeneration. Eur. J. Neurosci..

[31] Yang Y., Yang X.F., Wang Y.P., Tian Q., Wang X.C., Li H.L. (2007). Inhibition of protein phosphatases induces transport deficits and axonopathy. J. Neurochem..

[32] Jellinger K.A. (2011). Cell death mechanisms in neurodegeneration. J. Cell Mol. Med.

[33] Buée L., Bussière T., Buée-Scherrer V., Delacourte A., Hof P.R. (2000). Tau isoforms, phosphorylation and role in neurodegenerative disorders. Brain Res. Rev..

[34] Lee V.M., Goedert M., Trojanowski J.Q. (2001). Neurodegenerative tauopathies. Annu. Rev. Neurosci..

[35] Hanger D.P., Hughes K., Woodgett J.R., Brion J.P., Anderton B.H. (1992). Glycogen synthase kinase-3 induces Alzheimer’s disease- like phosphorylation of tau: generation of paired helical filament epitopes and neuronal localisation of the kinase. Neurosci. Lett..

[36] Hanger D.P., Seereeram A., Noble W. (2009). Mediators of tau phosphorylation in the pathogenesis of Alzheimer’s disease. Exp. Rev. Neurother..

[37] Hernandez F., Gomez D.B.E., Fuster-Matanzo A., Lucas J.J., Avila J. (2010). GSK3: apossible link between beta amyloid peptide and tau protein. Exp. Neurol..

[38] Wang J.H., Kelly P.T. (1995). Postsynaptic injection of Ca^2+^/CaM induces synaptic potentiation requiring CaMKII and PKC activity. Neuron.

[39] Gong C.X., Shaikh S., Wang J.Z., Zaidi T., Grundke-Iqbal I., Iqbal K. (1995). Phosphatase activity toward abnormally phosphorylated tau: decrease in Alzheimer disease brain. J. Neurochem..

[40] Sheldahl L.C., Slusarski D.C., Pandur P., Miller J.R., Kühl M., Moon R.T. (2003). Dishevelled activates Ca^2+^ flux, PKC, and CamKII in vertebrate embryos. J. Cell Biol..

[41] Garrido J.L., Godoy J.,A., Alvarez A., Bronfman M., Inestrosa N.C. (2002). Protein kinase C inhibits amyloid β peptide neurotoxicity by acting on members of the Wnt pathway. FASEB J..

[42] Alvarez A.R., Godoy J.A., Mullendorff K., Olivares G.H., Bronfman M., Inestrosa N.C. (2004). Wnt-3a overcomes β-amyloid toxicity in rat hippocampal neurons. Exp. Cell Res..

[43] Verkhratsky A., Petersen O.H. (2002). The endoplasmic reticulum as an integrating signalling organelle: from neuronal signalling to neuronal death. Eur. J. Pharmacol..

[44] Bootman M.D., Lipp P., Berridge M.J. (2001). The organisation and functions of local Ca^2+^ signals. J. Cell. Sci..

[45] Haug L.S., Jensen V., Hvalby O., Walaas S.I., Ostvold A.C. (1999). Phosphorylation of the inositol 1,4,5-trisphosphate receptor by cyclic nucleotide-dependent kinases *in vitro* and in rat cerebellar slices *in situ*. J. Biol. Chem..

[46] DeSouza N., Reiken S., Ondrias K., Yang Y.M., Matkovich S., Marks A.R. (2002). Protein kinase A and two phosphatases are components of the inositol 1,4,5-trisphosphate receptor macromolecular signaling complex. J. Biol. Chem..

[47] Bennett M.K., Calakos N., Scheller R.H. (1992). Syntaxin: a synaptic protein implicated in docking of synaptic vesicles at presynaptic active zones. Science.

[48] Sutton R.B., Fasshauer D., Jahn R., Brunger A.T. (1998). Crystal structure of a SNARE complex involved in synaptic exocytosis at 2.4 A resolution. Nature.

[49] Mayford M., Bach M.E., Huang Y.Y., Wang L., Hawkins R.D., Kandel E.R. (1996). Control of memory formation through regulated expression of a CaMKII transgene. Science.

[50] Wang J.Z., Gong C.X., Zaidi T., Grundke-Iqbal I., Iqbal K. (1995). Dephosphorylation of Alzheimer paired helical filaments by protein phosphatase-2A and -2B. J. Biol. Chem..

[51] Paterson D.S., Trachtenberg F.L., Thompson E.G., Belliveau R.A., Beggs A.H., Darnall R. (2006). Multiple serotonergic brainstem abnormalities in sudden infant death syndrome. JAMA.

[52] Cannon M.J., Williams A.D., Wetzel R., Myszka D.G. (2004). Kinetic analysis of beta-amyloid fibril elongation. Anal. Biochem..

[53] Hasselmo M.E., Sarter M. (2011). Modes and models of forebrain cholinergic neu-romodulation of cognition. Neuropsychopharmacology.

[54] Sacramento A.S., Moreira F.T.C., Guerreiro J.L., Tavares A.P., Sales M.G.F. (2017). Novel biomimetic composite material for potentiometric screening of acetylcholine, a neurotransmitter in Alzheimer's disease. Mater. Sci. Eng. C Mater. Biol. Appl..

[55] Duan Y., Liao A.P., Kuppireddi S., Ye Z., Ciancio M.J., Sun J. (2007). beta-Catenin activity negatively regulates bacteria-induced inflammation. Lab. Invest..

[56] Sun J., Hobert M.E., Duan Y., Rao A.S., He T.C., Chang E.B. (2005). Crosstalk between NF-κB and β-catenin pathways in bacterial-colonized intestinal epithelial cells. Am. J. Physiol. Gastrointest. Liver Physioly.

[57] Xie Y.K., Ding D., Wang H.M., Kang C.J. (2015). A homologue gene of *β*-catenin participates in the development of shrimps and immune response to bacteria and viruses. Fish Shellfish Immunol..

[58] Chen K., Wu Y., Zhu M., Deng Q., Nie X., Li M. (2013). Lithium chloride promotes host resistance against *Pseudomonas aeruginosa* keratitis. Mol. Vis..

[59] Baril M., Es-Saad S., Chatel-Chaix L., Fink K., Pham T., Raymond V.A. (2013). Genome-wide RNAi screen reveals a new role of a WNT/CTNNB1 signaling pathway as negative regulator of virus-induced innate immune responses. PLOS Pathog..

[60] Neumann J., Schaale K., Farhat K., Endermann T., Ulmer A.J., Ehlers S. (2010). Frizzled1 is a marker of inflammatory macrophages, and its ligand Wnt3a is involved in reprogramming *Mycobacterium tuberculosis*-infected macrophages. FASEB J..

[61] Undi R., Sarvothaman S., Narasaiah K. (2016). Toll-like receptor 2 signalling: significance in megakaryocyte development through wnt signalling cross-talk and cytokine induction. Cytokine.

[62] Gordon M.D., Dionne M.S., Schneider D.S., Nusse R. (2005). WntD is a feedback inhibitor of Dorsal/NF-kB in *Drosophila* development and immunity. Nature.

[63] Khush R., Leulier F., Lemaitre B. (2002). Lemaitre. Pathogen surveillance-the flies have it. Science.

