# Supplementary material for: Restrained Wnt Signaling Pathway by Enhanced EsGSK3β Activity Facilitates the Infection of Spiroplasma and Leads to Neuropathic Diseases in Crustaceans
Source: Mol Cell Proteomics. 2025 Aug 25;24(9):101059. doi: 10.1016/j.mcpro.2025.101059 (PMC12482308; doi:10.1016/j.mcpro.2025.101059)
Supplement: Supplementary Table 1 [file mmc1.docx]

**Table S2 The primers used in this paper.**

| Name | Sequence |
| --- | --- |
| Se-QF | ATACTCGTCAAACAGTATTTTTAAATTACTAT |
| Se-QR  Es*β*-Catenine-5’R1  Es*β*-Catenine-5’R2  Es*β*-Catenine-3’R1  Es*β*-Catenine-3’R2  Es*β*-Catenine-ORF-F  Es*β*-Catenine-ORF-R  EsGSK-3*β*-ORF-F  EsGSK-3*β*-ORF-R  Es*β*-Catenine-qF  Es*β*-Catenine-qR  EsGSK-3*β*-qF  EsGSK-3*β*-qR  Es*β*-Catenine-RNAiF  Es*β*-Catenine-RNAiR  EsGSK-3*β*-RNAiF  EsGSK-3*β*-RNAiR  Es*β*-Catenine-qiF  Es*β*-Catenine-qiR  EsGSK-3*β*-qiF  EsGSK-3*β*-qiR  EsALF1-qF  EsALF1-qR  EsALF2-qF  EsALF2-qR  EsALF3-qF  EsALF3-qR  EsCrustin-qF  EsCrustin-qR  EsHyastatin-qF  EsHyastatin-qR  EsSP-qF  EsSP-qR  EsLGBP-qF  EsLGBP-qR  EsSpaetzle-qF  EsSpaetzle-qR  EsGAPDH-F  EsGAPDH-R | AATCAATTGGTTTAAATAACCAAAGG  CATGGCGATCTGCTCTTTGGGGTTC  CCTCCTTGCCTGAGAGGGATGGCGC  CAAACTATGGCCCTATAGACCCCAG  GTGCCTGTGGACTCGATGCAGGGCC  GAATTCAGCTATCAGATGCCTCAGC  TGCGGCCGCCAGATCAGTGTCATACCAA  GAATTCATGAGTGGGCGACCCAGAA  TGCGGCCGCTCCCCTCTCTCCCTTTCCC  CGGTCTGTCAGGTTGGCGG  ACCTCTGCGTCTGGGTGCC  GCCGCAGGAGGTGTCATAC  ACCGCTTATCCTGGAGCAC  GCGTAATACGACTCACTATAGGGGAGACATGGCCGCCATGCCC  GCGTAATACGACTCACTATAGGCTGGCACGGGATGGCCTCACC  GCGTAATACGACTCACTATAGGAATATTGACGTGTGGAGCGC  GCGTAATACGACTCACTATAGGTGTTGGGGTCTTTCTCTTCC  CCCAGAAGGCCACTATGACCCC  GCATCGAGTCCACAGGCACATG  ATCGTACATCTGTTCCCGCTAC  GTTCATTTCCCGAATCTGCTC  GACGCAGGAGGATGCTAAC  TGATGGCAGATGAAGGACAC  GACCCTTTGCTGAATGCTTGA  CTGCTCTACAATGTCGCCTGA  ACGAGGAGCAAGGAAAGAAAG  TTGTGCCATAGACCAGAGACTT  GCTCTATGGCGGAGGATGTCA  CGGGCTTCAGACCCACTTTAC  ACGGTTCTAGACGCTCCTG  GGAAGTCGTAGCAGAGGTC  GGGTCATAACAACGTCAGGT  TCGAGTGGGAGACGGAGTT  CGACTCCACCCTCTTCATC  GTTGACGAGGTTGGTGGCA  GGAGACGTGTGTGAGGAAG  ATGATGGGGTTGCGGTTGC  CTGCCCAAAACATCATCCCATC  CTCTCATCCCCAGTGAAATCGC |
