# Supplementary material for: Restrained Wnt Signaling Pathway by Enhanced EsGSK3β Activity Facilitates the Infection of Spiroplasma and Leads to Neuropathic Diseases in Crustaceans
Source: Mol Cell Proteomics. 2025 Aug 25;24(9):101059. doi: 10.1016/j.mcpro.2025.101059 (PMC12482308; doi:10.1016/j.mcpro.2025.101059)
Supplement: Supplementary Table 2 [file mmc2.docx]

Table S1 Materials, reagents and instruments required for phosphoproteomic analysis.

| **Name** | **Company** | **Cat No.** |
| --- | --- | --- |
| Urea | Sigma | V900119-500G |
| Protease Inhibitor Cocktail III (protease inhibitor) | Merck Millipore | 539134-10ML |
| Protease Inhibitor Cocktail V (phosphatase inhibitor) | Merck Millipore | 539137-10VL |
| C18 ZipTips | Merck Millipore | / |
| BCA kit | Beyotime Biotechnology | P0011 |
| IMAC material | ThermoFisher Scientific | / |
| Trichloroacetic acid（TCA） | Sigma-Aldrich | T4885-2KG |
| Acetone | Hannuo Chemical | / |
| Triethylammonium bicarbonate buffer (TEAB) | Sigma-Aldrich | T7408-500mL |
| Trypsin | Promega | V5117 |
| Dithiothreitol (DTT) | Sigma-Aldrich | D9163-25G |
| iodoacetamide (IAM) | Sigma-Aldrich | V900335-5G |
| Trifluoroacetic acid（TFA） | Sigma-Aldrich | 302031-1L |
| Methanol | ThermoFisher Scientific | A452-4 |
| SPE strata-X100mg/3mL | phenomenex | 8B-S100-EBJ |
| TMT sixplex | ThermoFisher Scientific | 90068 |
| ACN (acetonitrile) | ThermoFisher Scientific | 204433 |
| TFA (trifluoroacetic acid) | Sigma-Aldrich | 302031-1L |
| TEAB (Triethylammonium bicarbonate buffer) | Sigma-Aldrich | T7408-500mL |
| Hydroxylamine | ThermoFisher Scientific | 90115 |
| ACN (acetonitrile) | ThermoFisher Scientific | 204433 |
| H_2_O | ThermoFisher Scientific | / |
| EASY-nLC 1000 UPLC system | ThermoFisher Scientific | EASY-nLC 1000 |
| Q Exactive Plus | ThermoFisher Scientific | Q Exactive Plus |
