# Supplementary material for: Restrained Wnt Signaling Pathway by Enhanced EsGSK3β Activity Facilitates the Infection of Spiroplasma and Leads to Neuropathic Diseases in Crustaceans
Source: Mol Cell Proteomics. 2025 Aug 25;24(9):101059. doi: 10.1016/j.mcpro.2025.101059 (PMC12482308; doi:10.1016/j.mcpro.2025.101059)
Supplement: Supplemental Figures [file mmc9.pdf]

Fig.S1

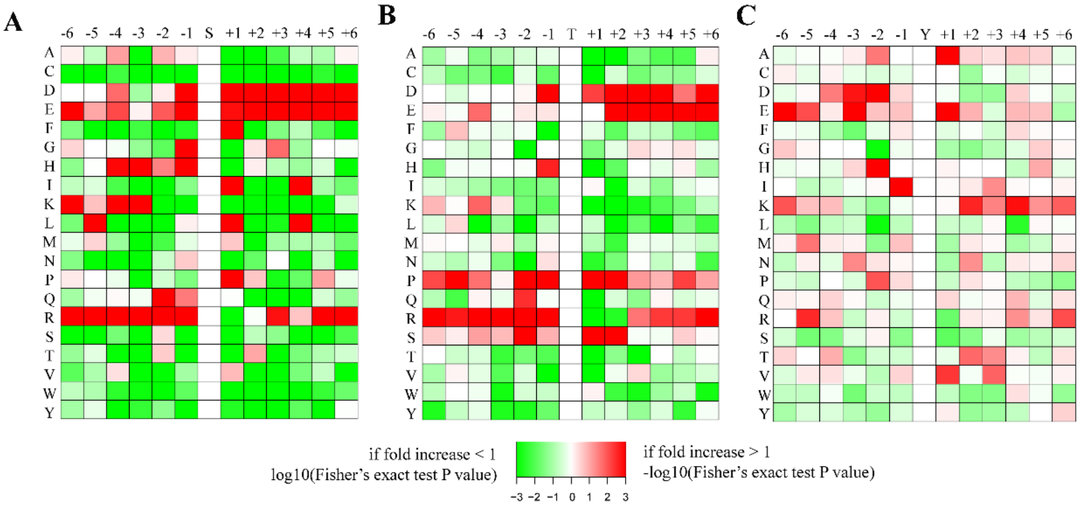

Figure S1. Motif analysis of all the identified sites against *E. sinensis* database.

Fig.S2

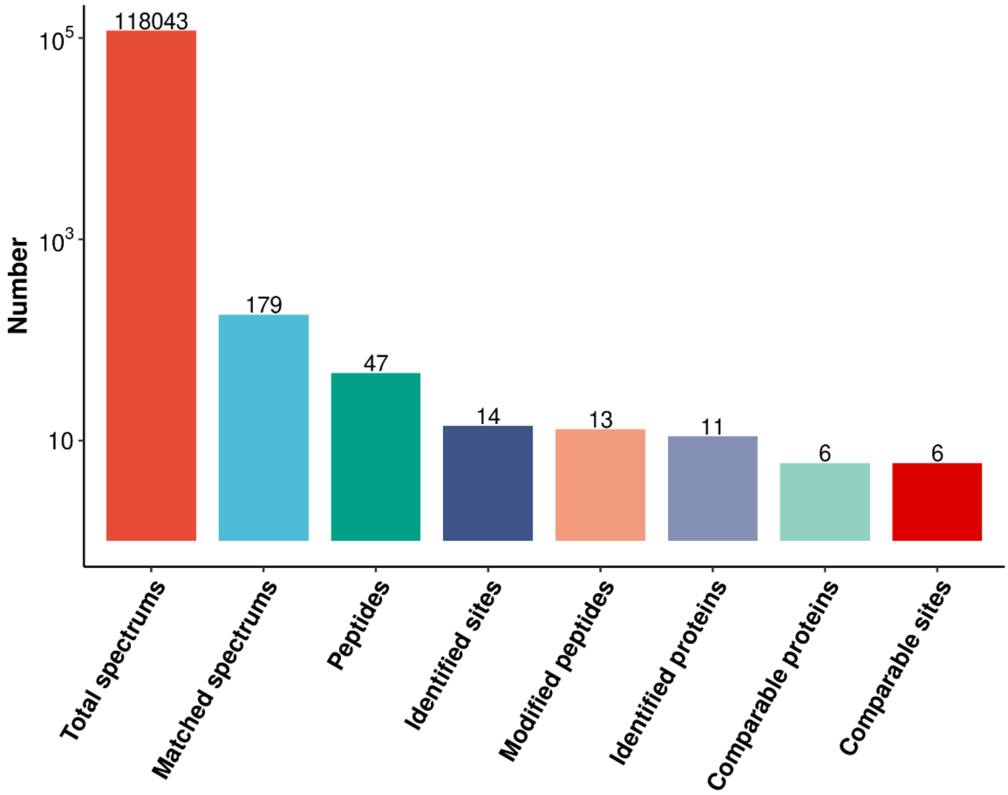

Figure S2. All the identified sites against *S. eriocheiris* database.

Fig.S3

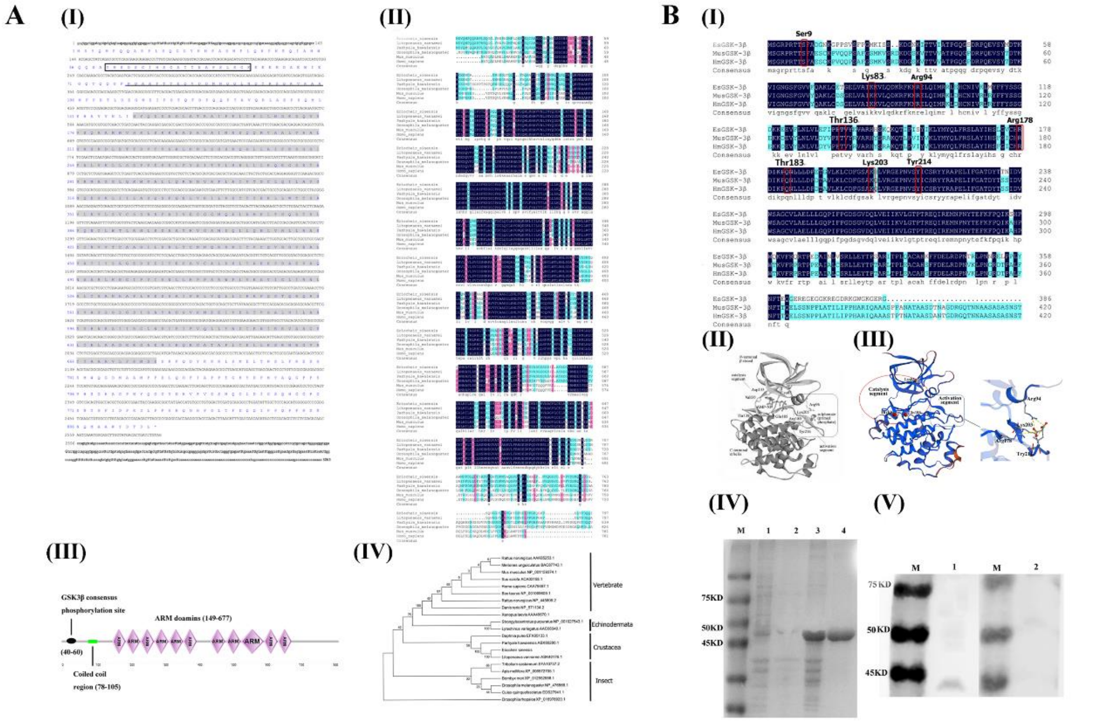

Figure S3. Sequence character analysis of the main components of the Wnt pathway. A. *β*-catenin information. **(I)** The full-length cDNA sequence and deduced amino acid sequences of Es*β*-catenin. The ORF of the nucleotide sequence is shown in uppercase letters, while the 5' and 3'-UTR sequences are shown in lowercase. The amino acid sequence was represented with one-letter codes above the nucleotide sequence. The GSK3*β* consensus phosphorylation site is boxed. The putative Armadillo/*β*-catenin-like repeat (ARM) domain is shaded. The coiled coil region is underlined. **(II)** Architecture and location representation of three characteristic domains of Es*β*-catenin. **(III)** Multiple sequence alignment of Es*β*-catenin. **(IV)** Phylogenetic tree analyses of Es*β*-catenin. **B.** GSK-3*β* information. **(I)** GSK3β sequence conservation analysis between *E. sinensis* and the corresponding protein in humans and mice. The red box is labeled as a highly conserved phosphorylation site; **(II)** The GSK3*β* structure of humans; **(III)** The GSK3*β* structure of *E. sinensis*; **(IV)** Expression and purification of the recombinant

protein EsGSK3*β*. Lanes M, molecular weight markers; Lanes 1 and 2 were soluble protein and insoluble protein of *E. coli* BL21 (DE3), respectively; Lane 3 was the insoluble protein of *E. coli* BL21 (DE3) and insoluble protein with pEt-28a after induction; and Lane 4 was the purified EsGSK3*β* protein; **(V)** The specificity detected by EsGSK3β polyclonal antibody. Lane M, molecular weight markers; Lane 1, *E. sinensis* hemocyte protein incubated with EsGSK3*β* polyclonal antibody; Lane 2, none (as a negative control).

Fig.S4

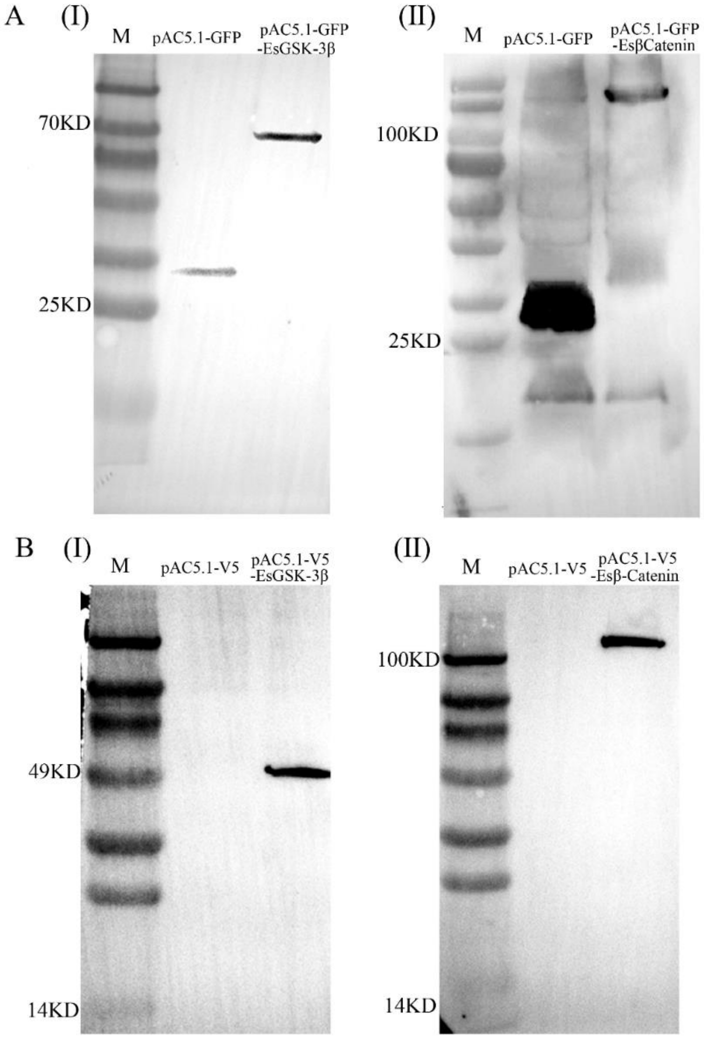

Figure S4. EsGSK3β and Es*β*-catenin over-expression in the S2 cell. (A) Western blot analysis the EsGSK3β-GFP and Esβ-catenin-GFP successfully expression in the S2 cell, pAc5.1-GFP as the control; **(B)** Western blot analysis the EsGSK3β-V5 and Esβ-catenin-V5

successfully expression in the S2 cell, pAc5.1-V5 as the control.
