# Supplementary material for: Restrained Wnt Signaling Pathway by Enhanced EsGSK3β Activity Facilitates the Infection of Spiroplasma and Leads to Neuropathic Diseases in Crustaceans
Source: Mol Cell Proteomics. 2025 Aug 25;24(9):101059. doi: 10.1016/j.mcpro.2025.101059 (PMC12482308; doi:10.1016/j.mcpro.2025.101059)
Supplement: Supplemental Material [file mmc10.docx]

**Supplemental Figure and Table legends**

**Table S1** The primers used in this paper.

**Table S2** Materials, reagents and instruments required for phosphoproteomic analysis.

**Table S3** Phosphorylated sites, peptides and proteins quantified against *E. sinensis* transcriptome data in the study.

**Table S4** The significantly changed (up-regulated and down-regulated) phosphorylated proteins identified in this study.

**Table S5** Phosphorylated sites, peptides and proteins quantified against *S. eriocheiris* Genomic data in the study.

**Table S6** KEGG pathway enrichment analysis the significantly changed (up-regulated and down-regulated) phosphorylated proteins identified in this study.

**Table S7** Domain enrichment analysis the significantly changed (up-regulated and down-regulated) phosphorylated proteins identified in this study.

**Table S8** KEGG enrichment analysis the differently expression genes obtained by transcriptome analysis.

**Figure S1.** Motif analysis of all the identified sites against *E. sinensis* database.

**Figure S2.** All the identified sites against *S. eriocheiris* database.

**Figure S3.** Sequence character analysis of the main components of the Wnt pathway. A. β-catenin information. (Ⅰ) The full-length cDNA sequence and deduced amino acid sequences of Esβ-catenin. The ORF of the nucleotide sequence is shown in uppercase letters, while the 5’ and 3’-UTR sequences are shown in lowercase. The amino acid sequence was represented with one-letter codes above the nucleotide sequence. The GSK3β consensus phosphorylation site is boxed. The putative Armadillo/β-catenin-like repeat (ARM) domain is shaded. The coiled coil region is underlined. (Ⅱ) Architecture and location representation of three characteristic domains of Esβ-catenin. (Ⅲ) Multiple sequence alignment of Esβ-catenin. (Ⅳ) Phylogenetic tree analyses of Esβ-catenin. B. GSK-3β information. (Ⅰ) GSK3β sequence conservation analysis between *E. sinensis* and the corresponding protein in humans and mice. The red box is labeled as a highly conserved phosphorylation site; (Ⅱ) The GSK3β structure of humans; (Ⅲ) The GSK3β structure of *E. sinensis*; (Ⅳ) Expression and purification of the recombinant protein EsGSK3β. Lanes M, molecular weight markers; Lanes 1 and 2 were soluble protein and insoluble protein of *E. coli* BL21 (DE3), respectively; Lane 3 was the insoluble protein of *E. coli* BL21 (DE3) and insoluble protein with pEt-28a after induction; and Lane 4 was the purified EsGSK3β protein; (Ⅴ) The specificity detected by EsGSK3β polyclonal antibody. Lane M, molecular weight markers; Lane 1, *E. sinensis* hemocyte protein incubated with EsGSK3β polyclonal antibody; Lane 2, none (as a negative control).

**Figure S4.** EsGSK3β and Esβ-catenin over-expression in the S2 cell. (A) Western blot analysis the EsGSK3β-GFP and Esβ-catenin-GFP successfully expression in the S2 cell, pAc5.1-GFP as the control; (B) Western blot analysis the EsGSK3β-V5 and Esβ-catenin-V5 successfully expression in the S2 cell, pAc5.1-V5 as the control.
